# IKKε phosphorylates kindlin-2 to induce invadopodia formation and promote colorectal cancer metastasis

**DOI:** 10.7150/thno.40397

**Published:** 2020-01-16

**Authors:** Ge Liu, Yantao Bao, Chaohua Liu, Qinchang Zhu, Lin Zhao, Xiaopeng Lu, Qian Zhu, Yafei Lv, Feng Bai, He Wen, Yujie Sun, Wei-Guo Zhu

**Affiliations:** 1 Guangdong Key Laboratory of Genome Instability and Human Disease Prevention, Department of Biochemistry and Molecular Biology, Shenzhen University School of Medicine, Shenzhen 518055, China.; 2 Key Laboratory of Carcinogenesis and Translational Research, Ministry of Education, Beijing Key Laboratory of Protein Posttranslational Modifications and Cell Function, Department of Biochemistry and Molecular Biology, School of Basic Medical Sciences, Peking University Health Science Center, Beijing 100191, China.; 3 Department of Pharmacy, Shenzhen University School of Medicine, Shenzhen 518055, China.; 4 Department of Pathology, Shenzhen University School of Medicine, Shenzhen 518055, China.; 5 Key Laboratory of Human Functional Genomics of Jiangsu Province, Nanjing Medical University, Nanjing, Jiangsu 210029, China.; 6 Carson International Cancer Center, Shenzhen University School of Medicine, Shenzhen 518055, China.

**Keywords:** IKKε, Invadopodia, Metastasis, Colorectal cancer, Kindlin-2 phosphorylation

## Abstract

Invadopodia formation is a key driver of cancer metastasis. The noncanonical IkB-related kinase IKKε has been implicated in cancer metastasis, but its roles in invadopodia formation and colorectal cancer (CRC) metastasis are unclear.

**Methods**: Immunofluorescence, gelatin-degradation assay, wound healing assay and transwell invasion assay were used to determine the influence of IKKε over-expression, knockdown and pharmacological inhibition on invadopodia formation and the migratory and invasive capacity of CRC cells *in vitro*. Effects of IKKε knockdown or pharmacological inhibition on CRC metastasis were examined in mice. Immunohistochemistry staining was used to detect expression levels of IKKε in CRC patient tissues, and its association with prognosis in CRC patients was also analyzed. Immunoprecipitation, western blotting and *in vitro* kinase assay were constructed to investigate the molecular mechanisms.

**Results**: IKKε co-localizes with F-actin and the invadopodia marker Tks5 at the gelatin-degrading sites of CRC cells. Genetic over-expression/knockdown or pharmacological inhibition of IKKε altered invadopodia formation and the migratory and invasive capacity of CRC cells *in vitro*. *In vivo*, knockdown or pharmacological inhibition of IKKε significantly suppressed metastasis of CRC cells in mice. IKKε knockdown also inhibited invadopodia formation *in vivo*. Clinical investigation of tumor specimens from 191 patients with CRC revealed that high IKKε expression correlates with metastasis and poor prognosis of CRC. Mechanistically, IKKε directly binds to and phosphorylates kindlin-2 at serine 159; this effect mediates the IKKε-induced invadopodia formation and promotion of CRC metastasis.

**Conclusions**: We identify IKKε as a novel regulator of invadopodia formation and a unique mechanism by which IKKε promotes the metastasis of CRC. Our study suggests that IKKε is a potential target to suppress CRC metastasis.

## Introduction

Colorectal cancer (CRC) is the second leading cause of cancer-related death worldwide 1. The majority of cancer mortalities are due to metastasis, which is a complicated process that requires cancer cells to escape from the primary tumor, intravasate into the bloodstream and extravasate to form new tumors at distant sites 2. During these events, invasive cancer cells form invadopodia — specialized F-actin-based membrane structures with proteolytic activity — that can penetrate through the epithelial and endothelial basement membrane by degrading the extracellular matrix (ECM) 3, 4. Invadopodia locate on ECM-degraded sites in many cancer cells, as observed in cell lines derived from CRC and primary tumor cells from patients 5, 6.

The ability of cancer cells to form invadopodia correlates well with their invasive behavior both *in vitro* and *in vivo* 7, 8. Direct* in vivo* visualization and characterization of invadopodia formation and function in cancer cells during the metastasis (including invasion, intravasation and extravasation) strongly indicate the key functional role of invadopodia in cancer metastasis 9, 10. Invadopodia form in response to various microenvironmental signals such as growth factors, hypoxia and ECM proteins 3. A large numbers of structural proteins and regulatory proteins that regulate actin dynamics, including cortactin, wiskott-aldrich syndrome protein (WASp), actin-related protein 2/3 (Arp2/3) complex, Rho-GTPases and adaptor proteins, such as Tks4 and Tks5 are also required 3, 4, 11. Although invadopodia formation has been well characterized, the molecular mechanisms of its regulation are still unclear.

The serine/threonine kinase IκB kinase subunit epsilon (IKKε) is a non-cannonical IKK kinase family member that shares about ~27% similarity to the canonical members of the IKK family of protein kinases IKKα and IKKβ. IKKε was originally identified as a breast cancer oncogene and consistently, its expression is typically amplified in breast cancers 12. Elevated IKKε levels are also found in several other cancers, including glioma, pancreatic cancer and ovarian cancer 13-15. IKKε promotes tumorigenesis by activating several signaling pathways, such as NF-kB and JAK/STAT pathways 16, 17. In addition, IKKε exhibits oncogenic function by directly phosphorylating and inhibiting tumor suppressors, including cylindromatosis (CYLD) and Forkhead box O 3a (FOXO3a) 18, 19.

Although a growing body of evidence has implicated IKKε in cancer metastasis 20, 21, a role for IKKε in invadopodia formation has not been explored. Several other kinases and phosphorylation events have, however, been implicated in regulating invadopodia formation. For example, Src-mediated tyrosine phosphorylation of cortactin and Tks5 is a critical for the trigger of invadopodia formation 22, 23. The Abl family of non-receptor tyrosine kinase (Arg) also mediates epidermal growth factor (EGF)-induced cortactin phosphorylation, triggering actin polymerization in invadopodia, ECM degradation, and tumor cell invasion 24. Fermitin family homolog 2 (FERMT2, also known as kindlin-2 or Mig-2), is a focal adhesion protein that is associated with increased metastatic potential of several types of cancers, including hepatocellular carcinoma, prostate cancer and gastric cancers 25-29. Kindlin-2 has been found to localize in invadopodia and be phosphorylated at serine 159 residue (S159); this event contributes to invadopodia formation in breast cancer cells 30. The kinase responsible for kindlin-2 phosphorylation is currently unknown.

Here, we aimed to determine the role of IKKε in invadopodia formation and CRC metastasis. We first tested the effects of IKKε over-expression, knockdown and pharmacological inhibition on invadopodia formation, and the migratory and invasive capacities of CRC cells *in vitro* and *in vivo*. We then monitored the association between the IKKε expression, CRC metastasis and clinical outcomes in patients with CRC. Finally, we assessed whether IKKε might be involved in mediating kindlin-2 phosphorylation.

## Materials and methods

### Cell lines and reagents

Normal human colon epithelial CCD841 cells and human CRC cell lines, including HCT116, SW480, SW620, DLD-1, HT-29 and Lovo cells were obtained from the American Type Culture Collection (ATCC). The cells were cultured in Dulbecco's Modified Eagle's Medium (DMEM; HyClone, Thermo Fisher Scientific) supplemented with 10% fetal bovine serum (FBS; Gibco), 100 U/ml penicillin (M&C Gene Technology) and 100 μg/ml streptomycin (M&C Gene Technology) in a 37 ℃ incubator with a 5% CO_2_ and humidified atmosphere. The small molecular inhibitor including Amlexanox (S3648) was purchased from Selleck and the IKK-3 inhibitor (HY-14682) was purchased from MedChemExpress. A 50 mM stock solution of each inhibitor was prepared in dimethyl sulfoxide (DMSO; Sigma-Aldrich). Isopropy-β-D-thiogalactoside (IPTG) and neomycin were purchased from Sigma-Aldrich.

### Plasmids and RNAi

Full length kindlin-2 expression constructs were amplified and cloned into the p3×FLAG-CMV-10, pGEX-4T3 vector (Addgene). GFP-tagged IKKε and Myc-DDK-tagged IKKε were purchased from OriGene. Site-specific mutations in kindlin-2 (S159A and S159D) and IKKε (K38A) were generated using a site-directed mutagenesis kit (Vazyme). The following siRNAs were used to silence target genes by transient transfection with UltraFection 3.0 (4A Biotech) reagent, according to the manufacturer's instructions: IKKα-1: 5'-GCCUUACACAGCCACUGUUTT-3';

IKKα-2: 5'-GCAGCCAUUUACCUGGCAUTT-3';

IKKβ-1: 5'-GGAAAUGAAAGAGCGCCUUTT-3';

IKKβ-2: 5'-GGAAGUACCUGAACCAGUUTT-3';

IKKε-1: 5'-CCACUGCCAGUGUGUACAATT-3';

IKKε-2: 5'-CCUGCAUCCCGACAUGUAUTT-3';

TBK1-1: 5'-CCAUGUGGGAGUUUAUACATT-3';

TBK1-2: 5'-GCCUUCUGGUGCAAUAUCUTT-3'.

### Establishment of stable cell lines

#### Overexpression

Kindlin-2 overexpressing cells were generated by transfection of constructs containing the full length or specific mutant kindlin-2 sequences cloned into a p3×FLAG-CMV-10 vector using UltraFection 3.0 (4A Biotech). The transfected cells were selected in neomycin.

#### shRNA knockdown

Stable IKKε knockdown cells were generated by UltraFection 3.0 (4A Biotech) tranfection of shRNA constructs with the IKKε siRNA sequences cloned into a pGPU6/Neo vector (GenePharma). The transfected cells were selected in neomycin. shIKKε-1: 5'-CCACUGCCAGUGUGUACAATT-3'; shIKKε-2: 5'-CCUGCAUCCCGACAUGUAUTT-3'.

#### CRISPR-Cas9 gene editing

Kindlin-2 KO cells were generated by UltraFection 3.0 (4A Biotech) transfection of sgRNA constructs in a px459/Puro vector (Addgene) following the protocol as described previously 31. The following sgRNA sequences to target kindlin-2 were used: sgRNA-F: 5'-CACCGTCAGGGTGACATCGCGGTTC-3'; sgRNA-R: 5'-AAACGAACCGCGATGTCACCCTGAC-3'.

### Western blotting and antibodies

Proteins were analyzed by western blotting as previously described 32. The following antibodies were used in this study: anti-kindlin-2-S159 (CTM-152, PTM Biolabs) ; anti-kindlin-2 (K3269) and anti-FLAG (F3165) (Sigma-Aldrich); anti-IKKα (2682), anti-IKKβ (2678), anti-IKKε (2905), anti-TBK1 (3504) (Cell Signaling Technology); anti-GAPDH (sc-3223), anti-FISH (sc-376211) and anti-cortactin (sc-55579) (Santa Cruz); anti-IKKε (ab7891) (Abcam); anti-SH3PXD2A (18976-1-AP) (Proteintech); anti-MIG2/Kindlin-2 (NBP1-47745) (NOVUS).

### Immunoprecipitation assay

Cells were harvested and lysed in NP-40 buffer (20 mM Tris·HCl pH 8.0, 137 mM NaCl, 1% NP-40, 10% glycerol, 2 mM EDTA, 1% protease inhibitor cocktail) for 30 min on ice. The supernatant was collected and incubated with the indicated antibodies at 4 ℃ rotating overnight. After adding protein A/G agarose beads (GE Healthcare), the incubation was further continued for 3 h at 4 ℃. The beads were then washed and analyzed by western blotting.

### *In vitro* kinase assay

Constructs for GST-tagged wild-type kindlin-2 and kindlin-2 (S159A) were transformed to E.coli strain BL21 and induced with 0.1 mM IPTG (Sigma-Aldrich) overnight at 16 ℃ and then purified using glutathione-Sepharose 4B beads (GE Healthcare) as previously described 33. Myc-DDK-tagged IKKε (WT) and IKKε (K38A) were transfected into HEK293T cells. After incubation for 48 h, the IKKε (WT) and IKKε (K38A) proteins were immunoprecipitated overnight with FLAG-conjugated M2 agarose beads and then eluted with Flag peptide (Sigma-Aldrich). Recombinant kindlin-2 and recombinant IKKε were mixed in kinase buffer [10 mM Tris·HCl pH 7.4, 10 mM NaCl, 10 mM MgCl_2_, 0.5 mM DTT, phosphatase inhibitor (PhosSTOP)]. The reaction was initiated by adding 100 μM ATP and incubated at 30 ℃ for 2 h. After denaturation by adding 5×SDS/PAGE sample buffer and boiling at 100 ℃ for 5 min, the samples were analyzed by western blotting.

### Wound healing assay

Cells were seeded into 6-well plates and left to grow to confluency for around 24 h. Then the culture medium was replaced with DMEM medium without serum to minimize cell proliferation. The cells were then scratched with a pipette tip and cellular migration was observed and imaged under a microscopy.

### Transwell invasion assay

Cells were seeded into chambers with filters (pore size of 8 μm) coated with matrigel to monitor cell invasive capacity. Briefly, the cells were suspended in serum-free DMEM (3×10^5^ cells/well) and then loaded into the upper chamber, medium containing 10% FBS was added to the lower chambers. After a 48 h incubation, the cells in the upper chambers were removed. Invasive cells on the bottom surface of the filters were fixed with 4% paraformaldehyde (PFA) and stained with 0.05% crystal violet. Representative images were captured under a microscope, and the total number of invaded cells was quantified using Image J software (National Institutes of Health).

### Gelatin degradation assay

Glass-bottomed dishes were treated with 0.1 mg/ml Poly L-lysine (Sigma-Aldrich) and crosslinked with 0.5% glutaraldehyde (Mecoxlane). Then, the dishes were coated with fluorescent gelatin [1:5 dilution of Oregon Green 488 conjugated gelatin (G13186, Invitrogen) with 0.1% unconjugated gelatin (G1393, Sigma)] at 37 ℃ for 1 h. The dishes were then incubated with 5mg/ml sodium borohydride for 15 min, and then incubated with 70% ethanol for 20 min. Three washes with 1×PBS were performed between each step. DMEM media was added to the dishes at 37 ℃ for 1 h before cell plating. After incubating the cells at 37 ℃ for a further 48 h, the cells were fixed in 4% paraformaldehyde (PFA) and permeabilized with 0.5% Triton X-100, rinsed with PBS and blocked in 3% bovine serum albumin (BAS) in PBS. The cells were then incubated with primary antibodies against Tks5/FISH (sc-30122, Santa Cruz) and F-actin/Phalloidin (A12380, Invitrogen), and then incubated with Alexa Fluor 647-labelled secondary antibodies (A-21245, Invitrogen). The samples were observed under a Nikon A1 confocal microscope. Quantification of the degraded gelatin area was performed using Image J software (National Institutes of Health).

### Confocal microscopy analysis

Cells grown on a glass-bottomed dish were fixed with 4% PFA and permeabilized with 0.1% Triton X-100. Then the cells were blocked with 3% BSA and then incubated with the indicated primary antibodies in 1% BSA at 4 ℃ overnight. After three washes with PBS, the cells were incubated with a secondary antibody in 1% BSA for 2 h at room temperature in the dark and then washed three times with PBS. The samples were then exposed to F-actin/Phalloidin (A12380, Invitrogen) and observed under a Nikon A1 confocal microscope. Images and Z-stacks were acquired with NIS-Elements imaging software. Orthogonal views were generated in Imaris Software.

### Animal studies

Six-week-old female NOD-Prkdc^em26^Il2rg^em26^Nju (NCG) mouse (Model Animal Research Center, MARC) were used for *in vivo* experiments. HCT116 cells stably expressing IKKε-specific shRNA or control shRNA were transfected with pHBLV-luci control vector. Puromycin (Life Technologies) was used to select for stable transfectants as previously described 34. For orthotopic implantation experiment, HCT116 cells stably expressing luciferase and IKKε-specific shRNA or control shRNA (3×10^5^ cells in 30 μl) were injected into the submucosal layer of cecum. Following survival surgery, mice were allowed to recover and then returned to housing. The study continued for 35 days from the date of cell implantation and mice were imaged once each week using bioluminescence imaging. For tail vein infection experiment, HCT116 cells stably expressing luciferase and IKKε-specific shRNA or control shRNA (1×10^6^ cells in 100 μl) were injected into the lateral tail vein of mice. To evaluate whether IKKε inhibitor Amlexanox inhibits cancer cell metastasis *in vivo*, HCT116 cells stably expressing luciferase (1×10^6^ cells in 100 μl) were injected into the lateral tail vein of mice. The mice were treated with Amlexanox (50 mg/kg) or vehicle by oral gavage every other day. The study continued for 4 weeks from the date of cell implantation and mice were imaged once each week using bioluminescence imaging. For bioluminescence imaging, the mice were injected intraperitoneally with VivoGlo™ Luciferin (Promega) at a dose of 150 mg/kg body weight and bioluminescent images were examined by using an IVIS LuminaⅡ*in vivo* bioluminescence imaging system (Caliper Life Science). After euthanasia, the lungs, liver, kidneys and cecum of the mice were isolated to examine the intensity of the metastasis signals. The organs were fixed in 10% formalin, processed and then embedded in paraffin by standard techniques. Longitudinal sections were subjected to histomorphological analysis by via haematoxylin and eosin (H&E) staining or immunohistochemistry (IHC) staining. Paraffin sections of mouse samples were incubated with antibody against cytokeratin18 (Abcam, ab133263), EpCAM (Abcam, ab223582).All protocols used for animal studies were approved by the Institutional Animal Care and Use Committee of Shenzhen University (approval ID no.2018030101).

### Tissue microarray analysis and immunohistochemistry staining

The human CRC tissue microarrays containing tissue samples from 191 CRC patients (including 161 pairs of CRC tissue samples matched to their adjacent normal tissue samples) and the associated clinicopathological information were purchased from Shanghai Outdo Biotech. Co. Ltd. Antibodies against IKKε (ab7891, Abcam) and phospho-kindlin-2 (S159) (CTM-152, PTM Biolabs) were used for immunohistochemistry staining according to the manufacture's protocol. Each sample was assigned a score according to the intensity (no staining = 0; weak staining = 1, moderate staining = 2, strong staining = 3) and the extent of stained cells (0% = 0, 1-25% = 1, 26-50% = 2, 51-75% = 3, 76-100% = 4). The final immunoreactive score was determined by multiplying the intensity with the extent, ranging from 0 (the minimum) to 12 (the maximum). Tissue arrays were used according to Institutional Review Boards (IRB) protocols approved by Shenzhen University and Shanghai Outdo Biotech. Co. Ltd. All human subjects gave informed consent.

### Statistical analysis

The data represent the means±S.D. of at least three independent experiments. Statistical analyses were performed by GraphPad Prism 5. The differences between two groups were assessed by unpaired two-tailed Student's *t* tests. Multi-group comparisons were analyzed by one-way ANOVA with Tukey's post hoc test. Survival curves were derived from Kaplan-Meier estimates and survival analysis was performed using the log-rank test. Pearson's r test was applied to evaluate the correlations among the groups. *P*<0.05 was considered statistically significant (*P<0.05, **P< 0.01, and ***P < 0.001).

## Results

### IKKε is required for invadopodia formation and ECM degradation

**Figure 1:**
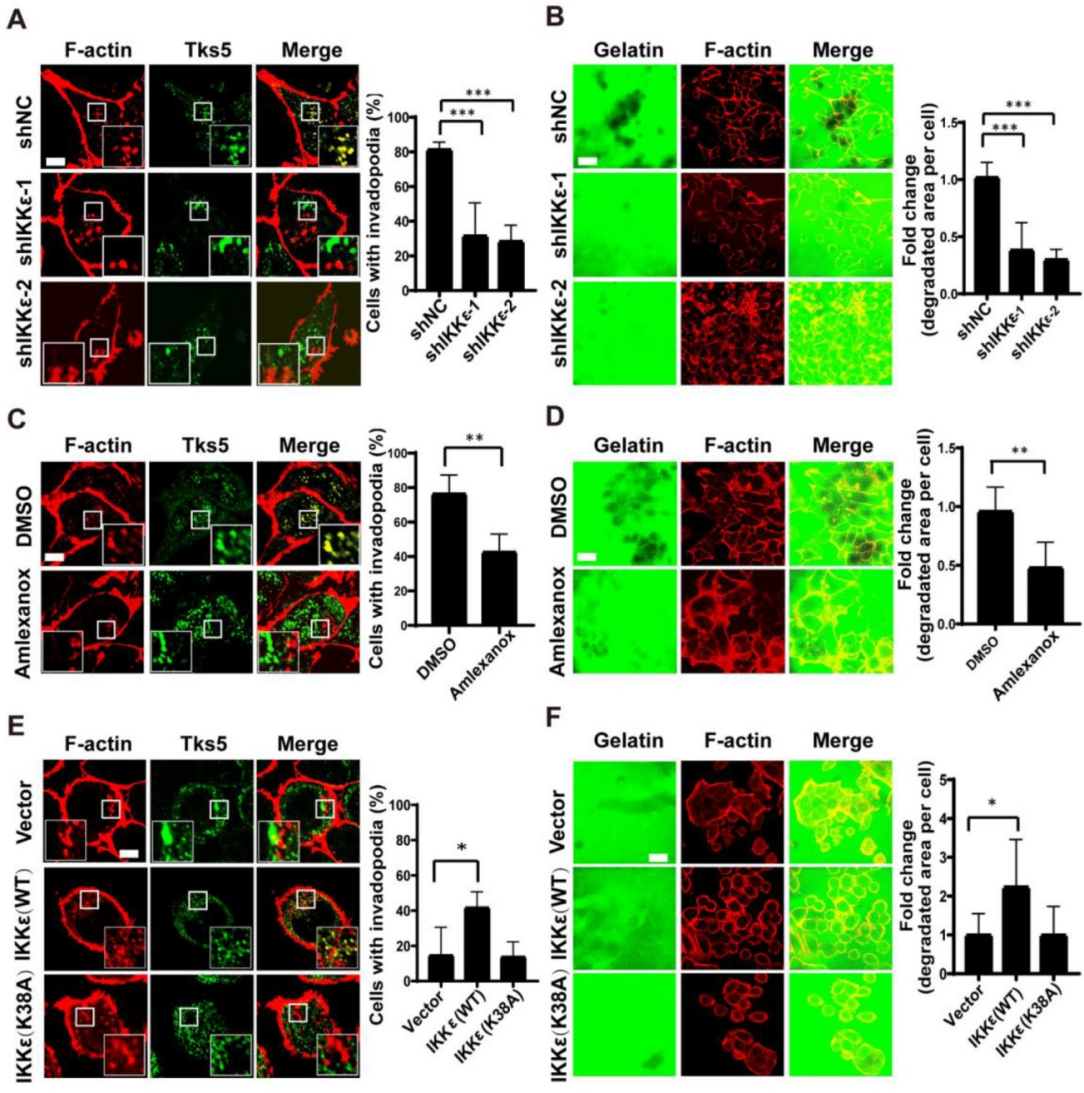
** IKKε induces invadopodia formation and ECM degradation.** (A) Tks5 and F-actin co-localization in HCT116 cells stably transfected with non-targeting shRNA or shRNA targeting IKKε (left). Inset shows magnified image of invadopodia in the box. Quantification of percentage of cells with invadopodia (right). (B) Gelatin degradation in HCT116 cells stably transfected with non-targeting shRNA or shRNA targeting IKKε (left). Quantification of the area of degraded gelatin per cell (right). (C) Tks5 and F-actin co-localization in HCT116 cells treated with DMSO or Amlexanox (100 μM) (left). Inset shows magnified image of invadopodia in the box. Quantification of percentage of cells with invadopodia (right). (D) Gelatin degradation in HCT116 cells treated with DMSO or Amlexanox (100 μM) (left). Quantification of the area of degraded gelatin per cell (right). (E) Tks5 and F-actin co-localization in SW480 cells stably transfected with vector, wild-type IKKε (WT) or mutant IKKε (K38A) (left). Inset shows magnified image of invadopodia in the box. Quantification of percentage of cells with invadopodia (right). (F) Gelatin degradation in SW480 cells stably transfected with vector, wild-type IKKε (WT) or mutant IKKε (K38A) (left). Quantification of the area of degraded gelatin per cell (right). All data represent the means±S.D. of three independent experiments (N>200 cells/sample, *P<0.05, **P< 0.01, and ***P < 0.001).

We hypothesized that IKKε might have a role in regulating invadopodia formation and ECM degradation. To test our hypothesis, we first monitored the effects of silencing endogenous IKKε in the highly metastatic human CRC HCT116 cell line. We silenced IKKε by stable transfection of two different IKKε-targeted shRNA (shIKKε-1 and shIKKε-2), and compared the effects to cells transfected with a non-targeted, scrambled control (shNC). We first quantified the number of cells (plated on a gelatin matrix) with invadopodia based on the co-localization of the invadopodia markers, Tks5 and F-actin. We then determined ECM degradation by measuring the area of fluorescently labeled gelatin degraded per cell. After efficient endogenous IKKε knockdown (Figure S1 A), we observed significant decreases (>50%) in the number of cells forming invadopodia compared to shNC cells (Figure 1 A). Consistent with these decrease in the number of invadopodia-forming cells, decreases (approximately 3 fold) in the area of gelatin degraded per cell in shIKKε cells were also observed (Figure 1 B).

We next treated the cells with an IKKε-specific kinase inhibitor amlexanox 35 to measure the influence of inhibiting IKKε kinase activity on invadopodia formation and ECM degradation. Again, we observed a reduced number of cells forming invadopodia in cells treated with amlexanox (100 μM) compared to DMSO-treated controls (42% vs.78%) (Figure 1C). The area of gelatin degraded per cell was also significantly decreased (approximately 2 fold) in cells treated with amlexanox when compared to controls (Figure 1D). These data confirmed that specifically inhibiting IKKε kinase activity could inhibit invadopodia formation and ECM degradation.

Finally, we tested whether IKKε is sufficient to promote invadopodia formation and matrix degradation. SW480 CRC cells derive from Duck's B staging cancer with lower invasive ability, while HCT116 CRC cells derive from Duck's C staging cancer with higher invasive ability. Here, the endogenous levels of IKKε in SW480 CRC cells were found to be lower than that in HCT116 CRC cells (Figure S1 B). Consistently, lower invadopodia formation ability was observed in SW480 CRC cells when compared to HCT116 CRC cells (15% vs. ~80%) (Figure 1 A and E).Then we overexpressed Myc-DDK-tagged wild-type (WT) IKKε or kinase-dead IKKε mutant (K38A) 18 in SW480 cells (Figure S1 C). IKKε (WT) but not K38A mutant overexpression significantly resulted in an increase in the number of cells forming invadopodia (40%) compared to control cells (15%) (Figure 1E) and an increase (approximately 2 fold) in the area of degraded gelatin per cell (Figure 1F). Together, these results suggest that IKKε is required for invadopodia formation and its kinase activity contributes to the invadopodia formation in CRC cells.

### IKKε colocalizes with F-actin and Tks5 in invadopodia

**Figure 2:**
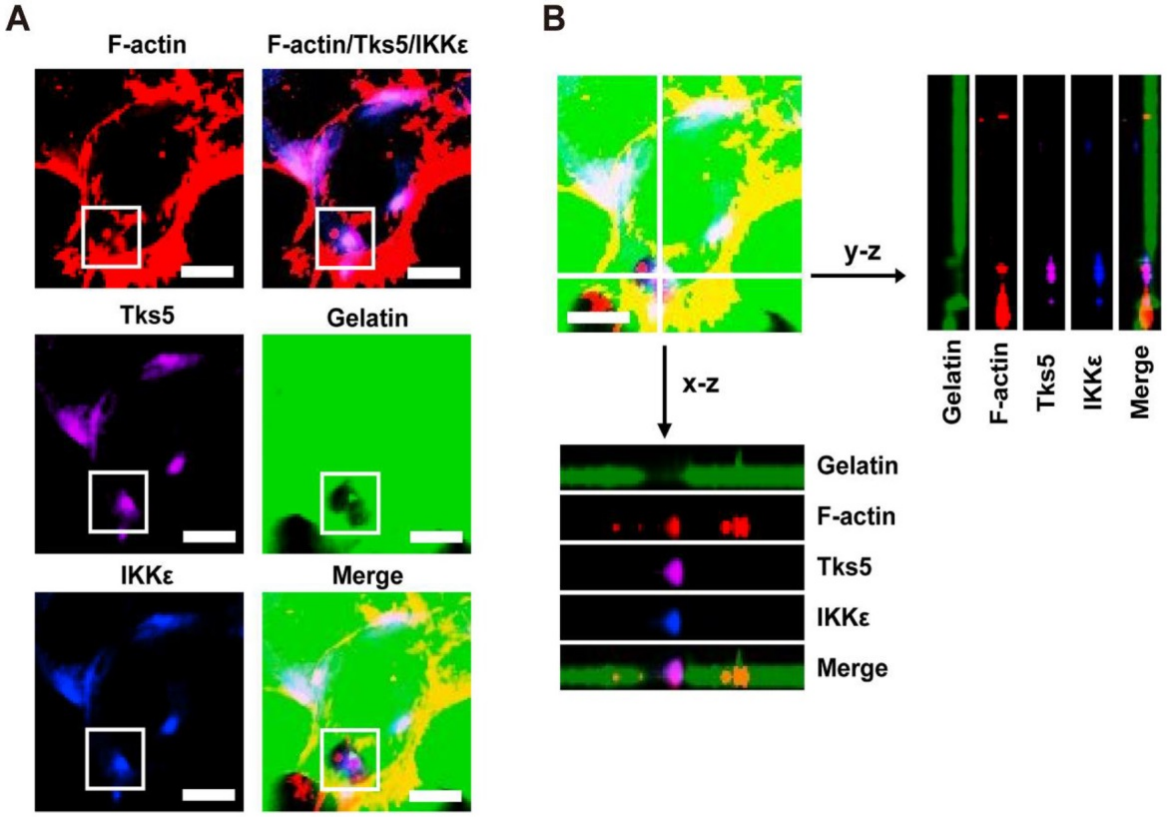
** IKKε co-localizes with F-actin and Tks5 in active invadopodia.** (A) F-actin (red), Tks5 (purple) and IKKε (blue) co-localization and gelatin (green) degradation in HCT116 cells. Inset highlight area of gelatin degradation where F-actin, Tks5 and IKKε are co-localized in the box. (B) Z-stack acquisition was performed and orthogonal views of the x-z plane and y-z plane are presented. Black arrows indicate areas of gelatin degradation where IKKε, Tks5 and F-actin co-localize. Scale bar, 10μm.

In light of the observations that IKKε promotes invadopodia formation and ECM degradation, we next wanted to know whether IKKε is locolized to invadopodia. Because F-actin co-localization with the unique adaptor protein Tks5 can be used to identify invadopodia, we again plated HCT116 cells onto Alex Fluor 488-conjugated gelatin and immunostained the cells with antibodies against IKKε, F-actin and Tks5. As shown in figure 2A, endogenous IKKε co-localized with Tks5 at gelatin-degradation puncta (black hole) that were rich of F-actin. We also observed co-localization of IKKε, Tks5 and F-actin throughout the degraded gelatin area by z-stack projection imaging (Figure 2B). These results demonstrate that IKKε localizes with F-actin and Tks5 in active invadopodia in CRC cells.

### IKKε promotes CRC cells migration and invasion *in vitro*

**Figure 3:**
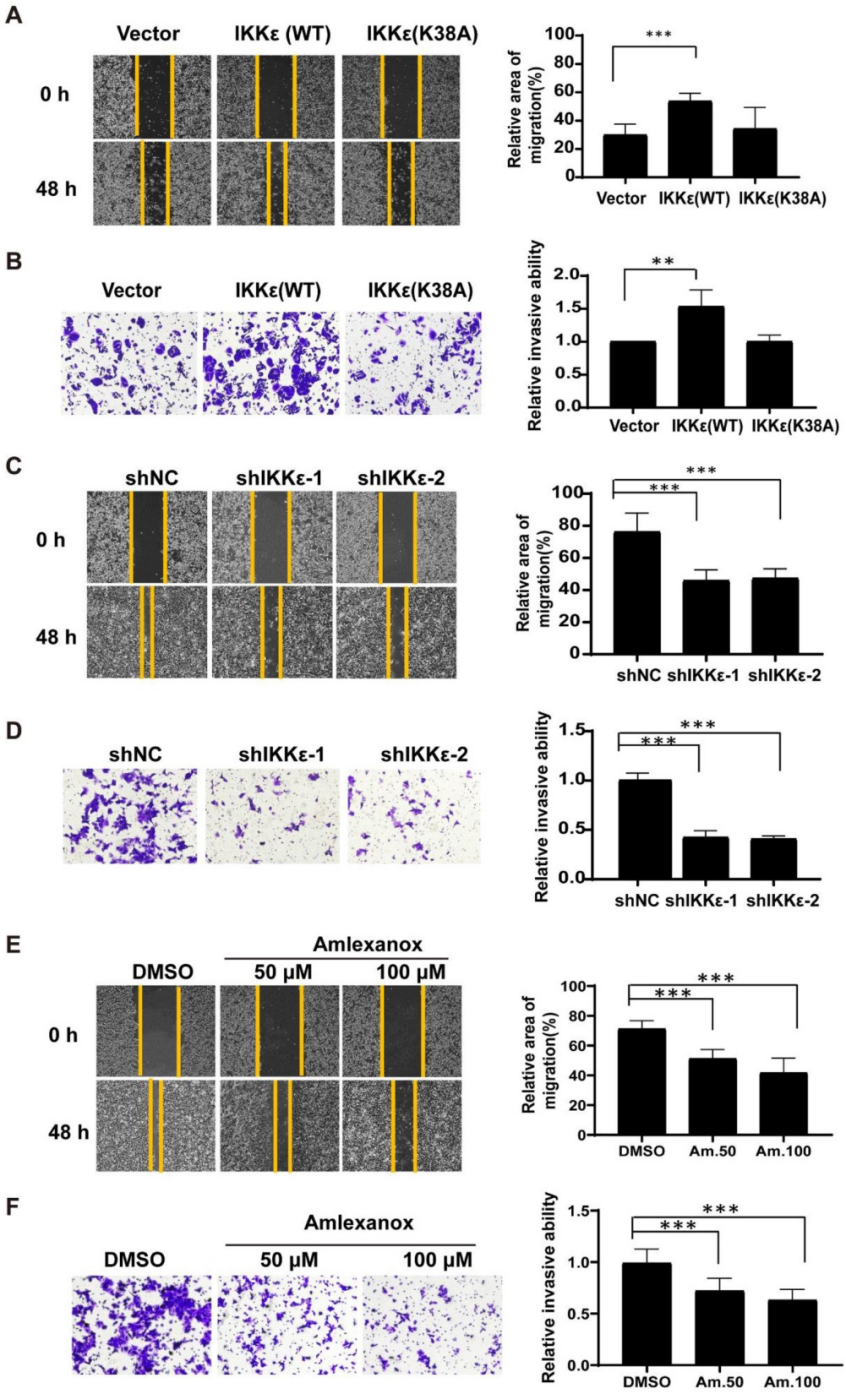
** IKKε promotes the migratory and invasive ability of colorectal cancer cells.** (A, B) Wound healing assay (A) and Transwell invasion (B) assays for SW480 cells stably transfected with vector, wild-type IKKε (WT) or mutant IKKε (K38A) (left). (A) The wound closure rate was determined as a percentage of the original wound area (right). (B) The relative invasive ability was normalized to vector (right). (C, D) Wound healing (C) and Transwell invasion (D) assay for HCT116 cells stably transfected with non-targeting shRNA or shRNA targeting IKKε (left). (C) The wound closure rate was determined as a percentage of the original wound area (right). (D) The relative invasive ability was normalized to shNC (right). (E, F) Wound healing (E) and Transwell invasion (F) assay for HCT116 cells treated with Amlexanox at the indicated concentrations (left). (E) The wound closure rate was determined as a percentage of the original wound area (right). Am. 50: amlexanox 50 μM; Am. 100: amlexanox 100 μM. (F)The relative invasive ability was normalized to DMSO (right). Am. 50: amlexanox 50 μM; Am. 100: amlexanox 100 μM. The cell number was counted in five randomly captured images for each sample. All data represent the means±S.D. of three independent experiments (**P< 0.01, and ***P < 0.001).

We next wanted to see whether IKKε had a role in mediating CRC cell migration and invasion. Here, we established wound healing and the transwell invasion assays with wild-type IKKε (WT) and kinase dead mutant IKKε (K38A) overexpressing SW480 cells and IKKε-knockdown HCT116 cells. As shown in Figure 3A, cells overexpressing IKKε (WT) but not IKKε mutant (K38A) showed a significant increase in the wound recovery (54%) compared to control cells (30%). Similarly, transwell invasion assay also showed that IKKε (WT) but not IKKε mutant (K38A) overexpression induced 1.5 fold-increase in the invasion of SW480 cells compared to the control cells (Figure 3B). Conversely, we observed dramatic reductions in wound recovery (approximately 2 fold) and invasion (approximately 2 fold) of the cells in IKKε-knockdown HCT116 cells compared to shNC cells (Figure 3 C and D). Assays with another CRC cell line Lovo cell have also been performed. We generated Lovo cells that stably expressing IKKε-specific shRNA or control shRNA, and evaluated their migration and invasion ability by transwell assays. As shown in supplemental figure S2, two shRNA sequences were used to perform the knockdown experiments. Both two shRNA sequences effectively down-regulated the expression of IKKε (Figure S2 A). Knockdown of IKKε significantly inhibited the migration and invasion of Lovo cells (Figure S2 B). These results are well consistent with that from HCT116 cells (Figure 3 C and D). Finally, HCT116 cells treated with amlexanox also showed a marked reduction in the migratory (Figure 3 E) and invasive capacity of HCT116 cells (Figure 3 F), in a dose-dependent manner (Figure 3 F). Together, these results demonstrate that IKKε promotes CRC cells migration and invasion.

### IKKε knockdown or inhibition suppress CRC cells metastasis and invadopodia formation *in vivo*

**Figure 4:**
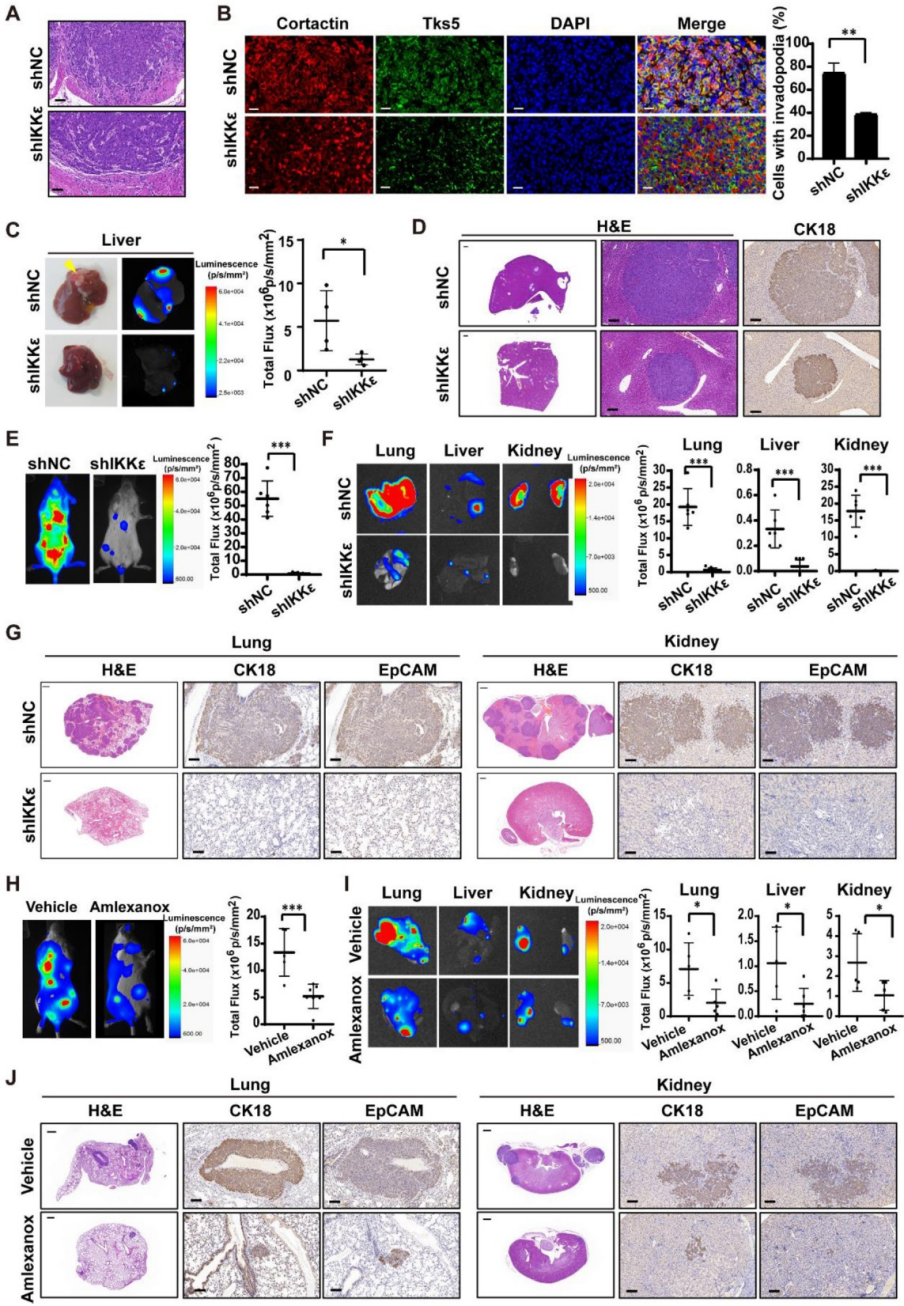
** IKKε knockdown or inhibition suppressed colorectal cancer cells metastasis and invadopodia formation *in vivo.***(A) H&E staining of the cecum derived from mice orthotopically microinjected with HCT116 cells stably expressing shNC or shIKKε. Scale bar, 50 μm. (B) Immunofluorescent analysis of Tks5 and cortactin co-localization in cecum tumor sections from shNC and shIKKε mice (left). Quantification of cells with invadopodia (right). Scale bars represent 200 μm. (N>300 cells/sample, **P<0.01,). (C) The gross view of tumor nodules established in liver and the bioluminescence images of liver from mice orthotopic microinjected with HCT116 cells stably expressing shNC or shIKKε (left). Yellow arrowheads indicate tumor nodules in the liver. Quantitative analysis of photon flux in shNC (n=4) and shIKKε (n=4) mice (*P<0.05) (right). (D) H&E staining of the liver derived from mice which were orthotopic microinjected with HCT116 cells stably expressing shNC or shIKKε (left). Scale bar, 500 μm (left) and 50 μm (middle). Images for IHC staining of CK18 in in metastatic sites in liver (right) Scale bar, 50 μm. (E) Bioluminescence images of shNC and shIKKε mice (left). Quantitative analysis of photon flux in shNC (n=6) and shIKKε (n=8) mice (***P<0.001) (right). (F) Bioluminescence images of lungs, liver and kidneys isolated from shNC and shIKKε mice (left). Quantification of photon flux in lungs, livers and kidneys isolated from shNC (n=6) and shIKKε (n=8) mice (***P <0.001) (right). (G) H&E staining sections of the lung and kidney sections from shNC and shIKKε mice (left). Scale bar, 500 μm. Images for IHC staining of CK18 and EpCAM in metastatic sites in lung and kidney (middle and right). Scale bar, 50 μm. (H) Bioluminescence images of vehicle-treated or amlexanox-treated mice (left). Quantitative analysis of photon flux in vehicle-treated (n=6) and amlexanox-treated (50 mg/kg, n=8) mice (***P <0.001) (right). (I) Bioluminescence images of lungs, liver and kidneys isolated from vehicle-treated or amlexanox-treated mice (left). Quantification of photon flux in lungs, livers and kidneys isolated from vehicle-treated (n=6) and amlexanox-treated (n=8) mice (*P<0.05) (right). (J) H&E staining sections of the lung and kidney sections from vehicle-treated or amlexanox-treated mice (left). Scale bar, 500 μm. Images for IHC staining of CK18 and EpCAM in in metastatic sites in lung and kidney (middle and right). Scale bar, 50 μm.

To confirm our findings* in vivo*, we generated HCT116 cells stably expressing luciferase and IKKε-specific shRNA or control shRNA. IKKε was almost completely knocked down in the cells stably expressing IKKε-specific shRNA (Figure S2 C). We injected HCT116 cells stably expressing luciferase and IKKε-specific shRNA (shIKKε) or control shRNA (shNC) into the submucosal layer of cecum of mice and examined primary tumors for histology. We found that tumor cells in shNC group invaded into surrounding stroma, while invasion of tumor cells in shIKKε group were inhibited (Figure 4 A). To clarify whether IKKε knockdown affects the invadopodia formation in CRC cells *in vivo*, we further examined invadopodia formation in the tumor cells in section of primary tumor tissues by staining with invadopodia markers including cortactin and Tks5. We found that the number of cells with invadopodia in shIKKε group was significantly less than that in the shNC group (Figure 4B), which was well consistent with the invasion of the cells in the primary tumor. We also monitored the metastasis in liver by bioluminescence imaging. Compared with mice in the shNC group (n=4), mice in the shIKKε group (n=4) showed a marked reduction in fluorescent signals in liver (Figure 4 C). Consistent findings were produced by H&E staining analysis of stained sections, which showed that metastatic foci in livers of shIKKε mice were less frequent than in shNC mice (Figure 4 D). HCT116 cells generated metastases in the liver were confirmed by H&E staining and immunochemistry staining for cytokeratin18 (CK18) (Figure 4 D). These results demonstrated that IKKε knockdown significantly suppresses invadopodia formation and the process of CRC cell invasion into microenvironment *in vivo*.

We also inoculated these cells into circulation through the tail vein infection to confirm the effect of IKKε knockdown on metastasis. At four weeks after the injection of CRC cells, we monitored metastatic dissemination of HCT116 cells by bioluminescence imaging. We detected a strong fluorescent signal (55.1×10^6^ p/s/mm^2^) throughout the bodies of mice carrying shNC control group (n=6), and a much weaker fluorescent signal (0.8×10^6^ p/s/mm^2^) in mice carrying shIKKε group (n=8) (Figure 4 E). Specifically, strong fluorescent signals were clearly detected in the lungs, liver and kidneys of mice carrying shNC group but not mice carrying shIKKε cells (Figure 4 F), indicating that IKKε knockdown decreases metastatic foci formation in these organs. Consistent findings were produced by H&E analysis of stained sections, which showed that metastatic foci in lungs and kidneys of shIKKε mice were less frequent than in shNC mice (Figure 4 G). HCT116 cells generated metastases in the lungs and kidneys were confirmed by immunochemistry staining with CK18 and epithelial cell adhesion molecule (EpCAM) (Figure 4 G). These data demonstrated that IKKε knockdown significantly suppresses CRC metastasis *in vivo*.

To further determine the effects of pharmacological inhibition of IKKε on cell metastasis *in vivo*, we injected HCT116 cells stably expressing luciferase into the tail vein of NCG mice and treated the mice with vehicle or amlexanox (50 mg/kg) by oral gavage. Compared with vehicle-treated mice (n=6), amlexanox-treated mice (n=8) showed a marked reduction in fluorescent signals throughout the bodies, as well as the lungs, liver and kidneys of mice (Figure 4 H and I). We also observed significant less metastatic foci in lungs and kidneys of amlexanox-treated mice compared to vehicle-treated mice (Figure 4 J). HCT116 cells generated metastases in the lungs and kidneys were confirmed by immunochemistry staining with CK18 and EpCAM (Figure 4 J).These results suggested that IKKε specific inhibitor amlexanox effectively inhibited metastasis of CRC cells *in vivo*.

### High IKKε expression correlates with metastasis and poor prognosis in CRC

**Figure 5:**
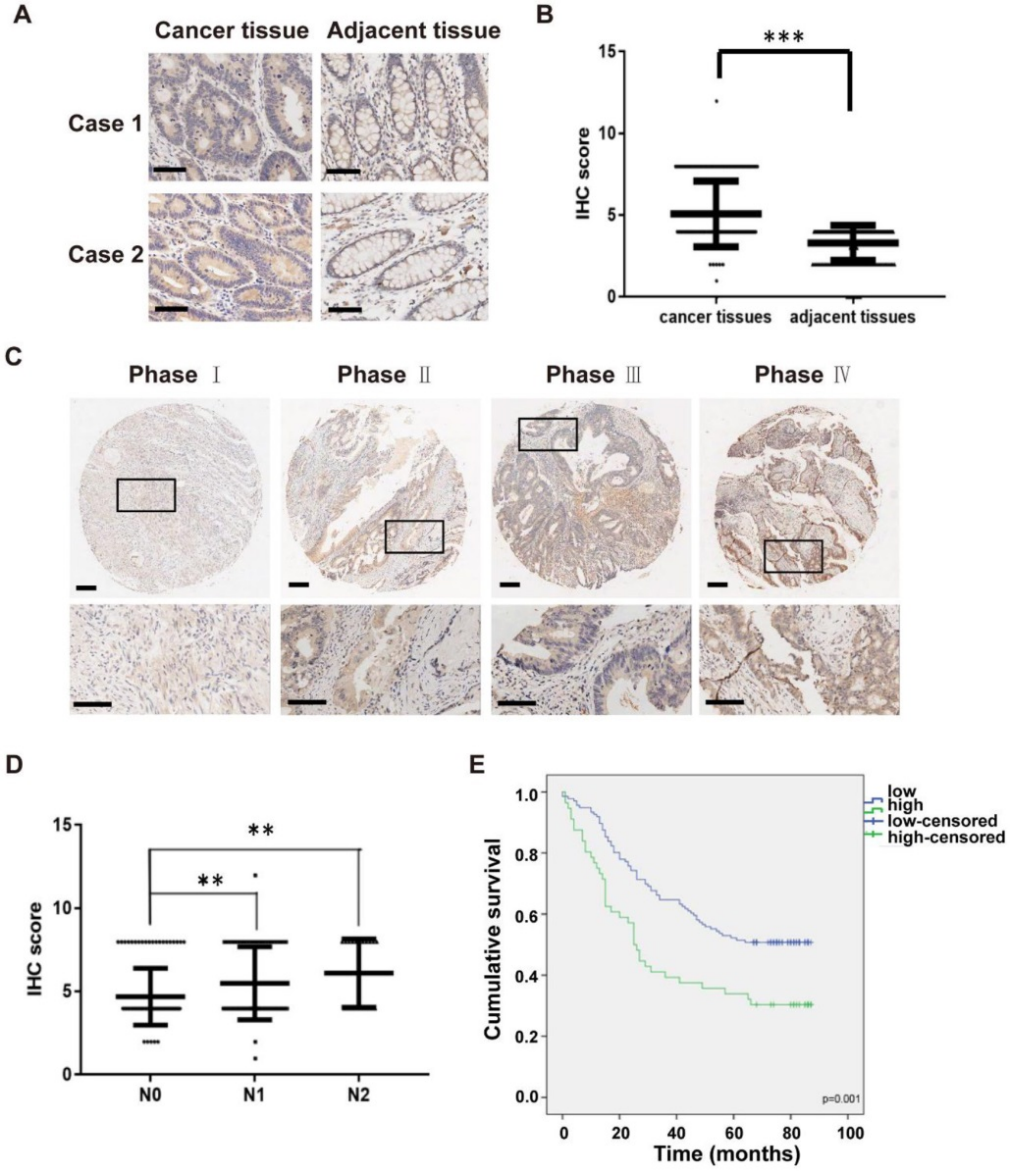
** High IKKε expression correlates with metastasis and poor prognosis in human colorectal cancer.** (A) Immunohistochemical staining for IKKε expression in human colorectal cancer tissues and paired adjacent tissues. Scale bar, 200 μm. (B) IKKε protein levels in 161 paired human colorectal cancer tissues and the adjacent normal tissues, measured by immunohistochemical analysis (***P <0.001). (C) Immunohistochemical staining for IKKε expression in human colorectal cancer tissue. Scale bar, 200 μm (top) and 100 μm (bottom). (D) IKKε protein levels in cancer tissues according to lymph node metastasis. (N classification) (**P<0.01). (E) Kaplan-Meier analysis of overall survival in a set of 191 colorectal cancer patients according to IKKε expression (log-rank test, p=0.001).

To examine the clinical impact of IKKε in CRC, we next determined the association between IKKε expression and clinicopathological features in patients with CRC. Here, we analyzed IKKε expression in tumor and adjacent normal tissue samples isolated from 191 affected patients by immunohistochemistry staining. A total of 161 pairs of human malignant CRC tissues and adjacent normal tissues samples were included in the analysis. Compared with matched adjacent normal tissues, we found that IKKε expression was significantly higher in CRC tissues (Figure 5 A and B, *p*=0.0001). We also found a significant correlation between IKKε expression and tumor-node-metastasis (TNM stage, *p*=0.0002) as well as lymph node metastasis (N stage, *p*=0.001), but not with any patient demographic characteristics, such as age or sex (Table S1). The IKKε expression levels in cancer tissues were lightly associated with advanced cancer stage (Figure 5 C), and the IKKε expression levels in cancer tissues with lymph node metastasis (N1 and N2 stage) were significantly higher than in those without metastasis (N0 stage) (Figure 5 D). Moreover, Kaplan-Meier analysis indicated that patients with high IKKε expression in CRC tissues showed a shorter overall survival time than those with low IKKε expression (*p*=0.001, Figure 5 E). These results suggest that IKKε expression correlates with metastasis and poor prognosis in CRC.

### IKKε directly phosphorylates kindlin-2 at S159

**Figure 6:**
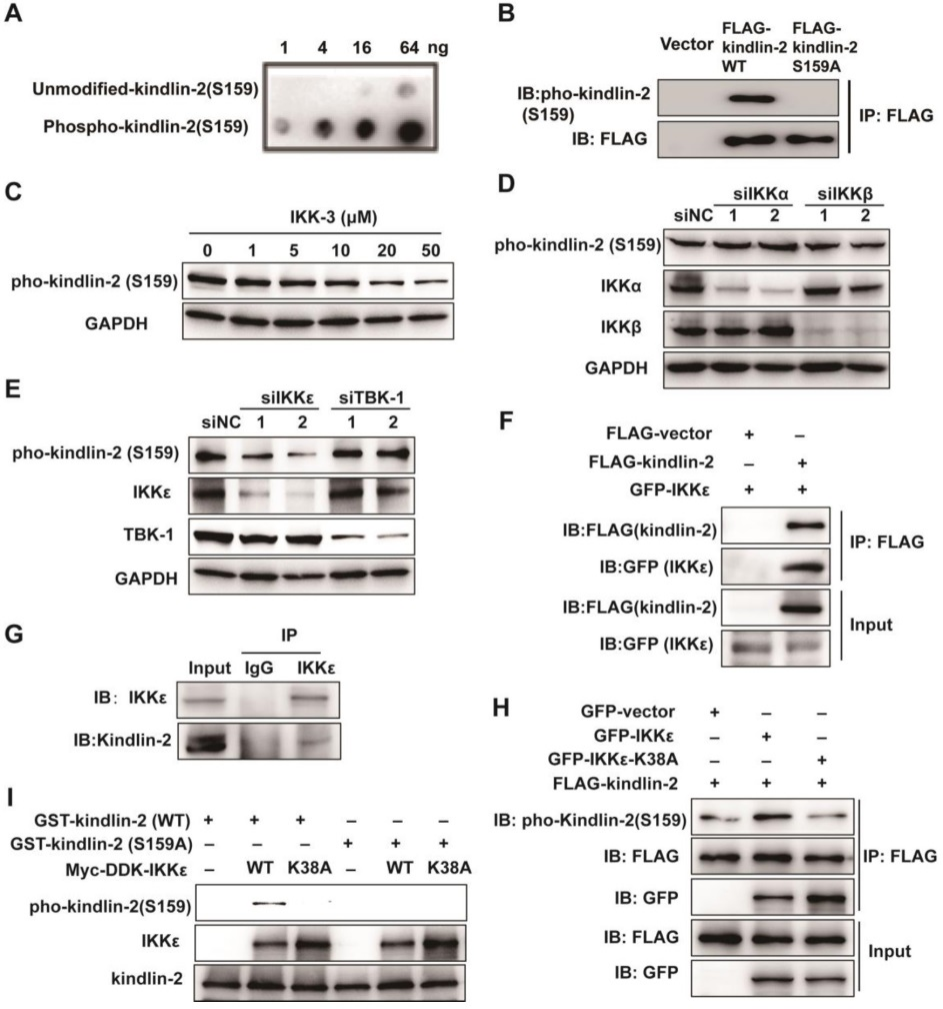
** IKKε directly phosphorylates kindlin-2 at S159.** (A) Slot blot analysis of the anti-phospho-kindlin-2-S159 antibody. The antibody was probed against an un-modified kindlin-2 peptide and phosphorylated peptides. (B) HCT116 cells were transfected with the indicated plasmids and whole cell extracts were immunoprecipitated (IP) with FLAG-conjugated M2 beads and then analyzed by immunoblotting (IB). (C)HCT116 cells were treated with the inhibitor IKK-3 at the indicated concentration for 24h and analyzed by immunoblotting. (D and E) HCT116 cells were transfected with the indicated siRNAs targeting IKKα, IKKβ, IKKε or TBK1. Cell lysates were analyzed by immunoblotting. (F) HCT116 cells were transfected with FLAG-tagged kindlin-2 and GFP-tagged IKKε for 48 h. Total cell lysates were immunoprecipitated using FLAG-conjugated M2 beads and analyzed by immunoblotting. (G) IKKε was immunoprecipitated using anti-IKKε antibody or the control antibody IgG from lysates of HCT116 cells. (H) HCT116 cells were transfected with the indicated plasmids and the whole cell extracts were prepared and analyzed by Co-IP assay flowed by immunoblotting with the indicated antibodies. (I) GST-tagged wild-type kindlin-2 or kindlin-2 (S159A) were incubated without IKKε (-), or with purified wild-type IKKε (WT) or mutant IKKε (K38A) in kinase buffer and resolved by SDS-PAGE, followed by immunoblotting analysis with the indicated antibodies.

Because a kinase-dead mutation of IKKε or pharmacological inhibition dramatically reduces the invadopodia formation, we speculated that the effects of IKKε on invadopodia formation rely on its kinase activity. As discussed, the focal adhesion protein kindlin-2 is phosphorylated at serine 159 and this phosphorylation event is thought to be involved in invadopodia formation 30. To determine whether the IKK protein family might phosphorylate kindlin-2, we generated a rabbit polyclonal antibody against the kindlin-2 S159 phosphopeptide (Figure 6 A and B) and used it to detect the changes in kindlin-2 phosphorylation in HCT116 cells treated with a pan-IKK inhibitor (IKK-3). IKK-3 reduced kindlin-2 phosphorylation levels at S159 in a dose-dependent manner (Figure 6 C), suggesting that the kinase that mediates kindlin-2 S159 phosphorylation does belongs to the IKK protein family.

To determine which kinase of the IKK family is involved in kindlin-2 S159 phosphorylation, we knocked down each major IKK family member by siRNA, including IKKα, IKKβ, IKKε and TBK1, and again monitored kindlin-2 phosphorylation levels. Kindlin-2 S159 phosphorylation was markedly reduced only when IKKε was knocked down (Figure 6 D and E), indicating that IKKε phosphorylates kindlin-2 at S159.

To verify whether IKKε and kindlin-2 interact, we performed a co-immunoprecipitation (co-IP) assay using transiently overexpressed GFP-tagged IKKε and FLAG-tagged kindlin-2 in HCT116 cells. Indeed, GFP-tagged IKKε interacted with FLAG-tagged kindlin-2 (Figure 6 F). We also detected an interaction between endogenous IKKε and kindlin-2 (Figure 6 G). Moreover, when GFP-tagged IKKε and FLAG-tagged kindlin-2 were co-expressed in the cells, we found that kindlin-2 S159 phosphorylation levels markedly increased (Figure 6H). Conversely, co-expression of the kinase-dead IKKε mutant (K38A) with FLAG-tagged kindlin-2 failed to increase kindlin-2 S159 phosphorylation levels (Figure 6 H).

Finally, we established an *in vitro* kinase assay, incubating purified kindlin-2 wild-type (WT) or phosphor-null mutant (S159A) mutant protein with wild-type IKKε (WT) or IKKε mutant (K38A). We found that only wild-type kindlin-2 was effectively phosphorylated by IKKε (WT) but not by IKKε (K38A) (Figure 6 I). Taken together, these results demonstrate that IKKε is the kinase responsible for phosphorylating kindlin-2 at S159.

### Kindlin-2 phosphorylation at S159 mediates IKKε-induced invadopodia formation in CRC

**Figure 7:**
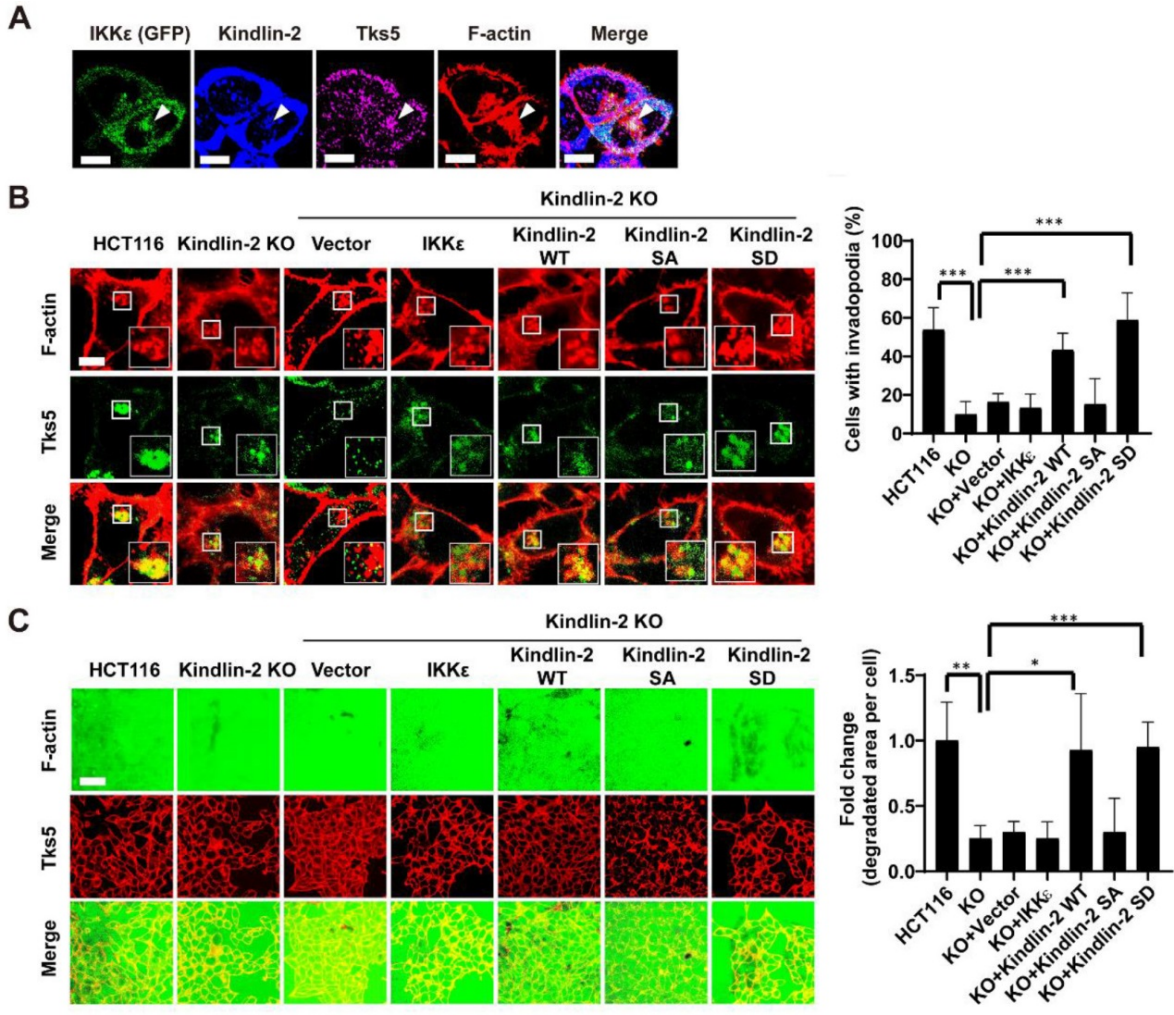
** IKKε-induced invadopodia formation depends on kindlin-2 S159 phosphorylation.** (A) HCT116 cells were transfected with GFP-tagged IKKε and stained with antibodies against kindlin-2, Tks5 and F-actin (phalloidin). Confocal images were acquired to visualize the localization of IKKε, kindlin-2, Tks5 and F-actin. White arrowhead point to the co-localization of IKKε, kindlin-2, Tks5 and F-actin. Scale bar, 10 μm. (B) Co-localization of Tks5 and F-actin in HCT116 cells with kindlin-2 knockout and rescue with wild-type IKKε, wild-type kindlin-2, phosphor-null mutant kindlin-2 (S159A) and phosphor-mimetic mutant (S159D) kindlin-2 (left). Inset shows magnified image of invadopodia in the box. Quantification of cells with invadopodia (right). (C) Gelatin degradation in HCT116 cells with kindlin-2 knockout and rescue with wild-type IKKε, wild-type kindlin-2, phosphor-null mutant kindlin-2 (S159A) and phosphor-mimetic mutant (S159D) kindlin-2 (left). The area of degraded gelatin per cell (right). All data represent the means ± S.D. (N>400 cells/sample, *P<0.05, **P< 0.01, and ***P < 0.001).

To determine whether IKKε-mediated kindlin-2 phosphorylation at S159 is responsible for IKKε-induced invadopodia formation and ECM degradation, we first investigated whether IKKε and kindlin-2 interact at invadopodia. We transfected GFP-tagged IKKε into HCT116 cells and then examined kindlin-2, IKKε, F-actin and Tks5 co-localization. Here, we found that IKKε clearly co-localized with kindlin-2 and Tks5, indicating that IKKε and kindlin-2 interacts at invadopodia (Figure 7A).

We next detected the expression levels of phosphorylated kindlin-2 (S159) in various CRC cell lines with different metastatic potential. We found that the high metastatic cell lines (Duck's C) including SW620, DLD-1, Lovo and HCT116 expressed high levels of phosphorylated kindlin-2 (S159) (Figure S3 A). But the low metastatic cell lines (Duck's B), including SW480 and HT-29, expressed low levels of phosphorylated kindlin-2 (S159) (Figure S3 A). Notably, almost non-detectable levels of phosphorylation of kindlin-2 (S159) were observed in normal colon epithelial cell line CCD841 (Figure S3 A). These results suggested that kindlin-2 (S159) phosphorylation was associated with the metastatic potential of CRC cells. Then, we generated a kindlin-2 knockout (KO) HCT116 cell lines, in which we knocked out kindlin-2 gene expression using CRISPR-Cas9 technology (Figure S3 B and C), and tested the effects on invadopodia formation and ECM degradation. Kindlin-2 KO led to a significant decrease in the number of cells with invadopodia and the area of gelatin degraded per cell (Figure 7B and C). After re-expressing wild-type (WT) or phosphor-mimetic mutant (S159D) kindlin-2, but not phosphor-null mutant kindlin-2 (S159A), we found that the number of cells with invadopodia and the area of gelatin degraded per cell was potently rescued (Figure 7 B and C). More importantly, overexpression of wild-type of IKKε in the kindlin-2 KO cells did not rescue invadopodia formation or ECM degradation (Figure 7 B and C). These results indicate that IKKε-mediated kindlin-2 phosphorylation at S159 is necessary and sufficient for IKKε-induced invadopodia formation.

**Figure 8:**
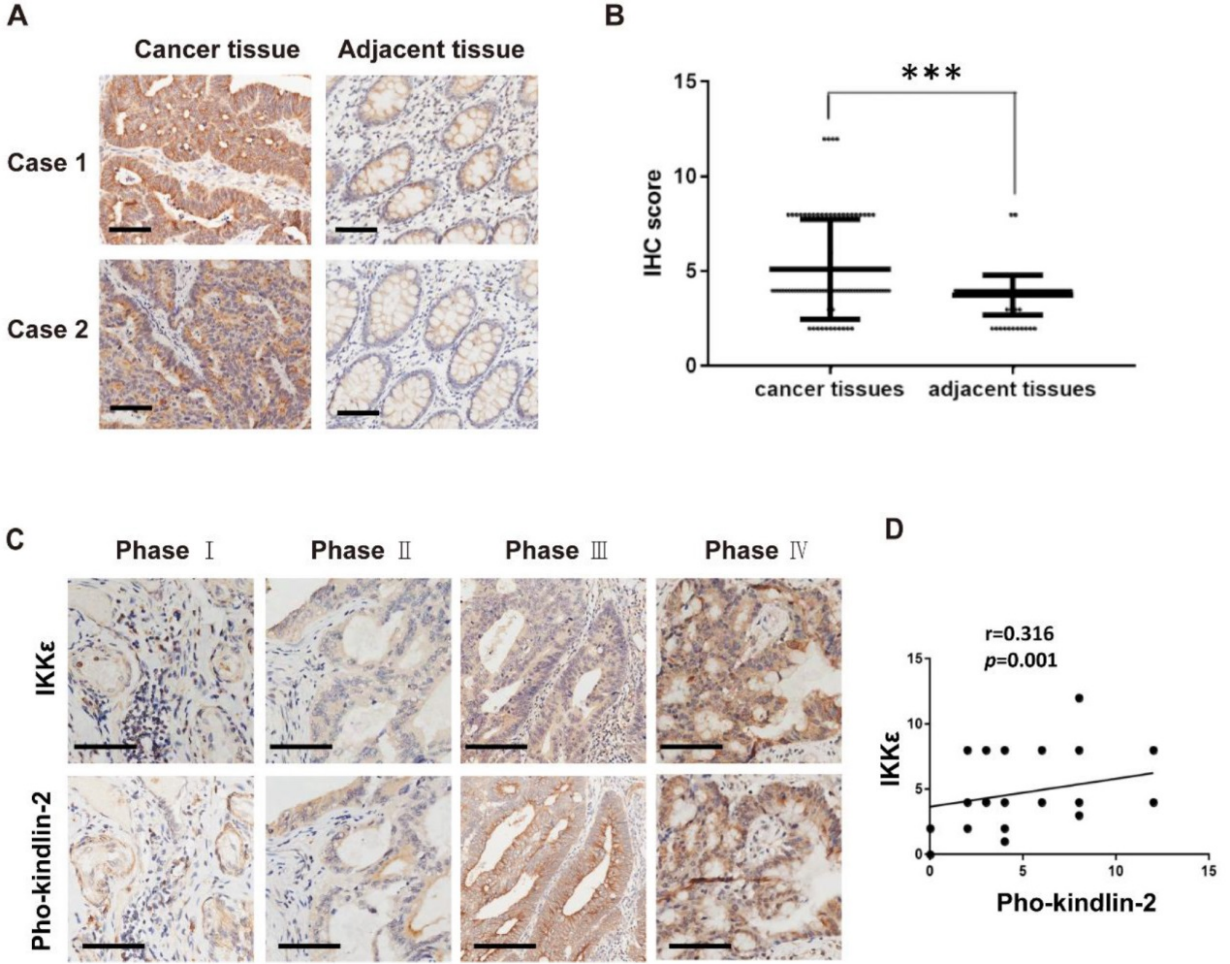
** Phosphorylated kindlin-2 (S159) levels positively correlate with IKKε expression in colorectal cancer tissues.** (A) Immunohistochemical staining for phosphor-kindlin-2-S159 in human colorectal cancer tissues and paired adjacent normal tissues. Scale bar, 200 μm. (B) Phosphor-kindlin-2-S159 protein levels in 161 paired human colorectal cancer tissues and adjacent normal tissues as measured by immunohistochemical analysis. The data represent the means ± S.D. (***P < 0.001). (C) Representative images of paraffin tissue sections stained with IKKε and phosphorylated kindlin-2 (S159) antibody. Scale bar, 200 μm. (D) Correlation analyses of IKKε and phosphorylated kindin-2 (S159) protein levels in human colorectal cancer specimens.

In our final analysis, we compared the expression of phosphorylated kindlin-2 (S159) in the tissues from the CRC patients by immunohistochemistry staining. We observed higher expression levels of phosphorylated kindlin-2 (S159) in CRC tissues compared with the matched adjacent normal tissues (*p*<0.001, Figure 8 A and B). We also assessed the association between IKKε and kindlin-2 phosphorylation in cancer tissues from CRC patients at different stages. Phosphorylated kindlin-2 (S159) levels positively correlated with IKKε expression in CRC tissues (n=161, r=0.316, *p*=0.001, Figure 8 C and D). These results from our clinical investigations provide further support for a direct and functional association between IKKε and kindlin-2.

## Discussion

Invadopodia formation is associated with tumor invasiveness and metastatic potential. Here we aimed to determine how invadopodia formation is regulated and how this regulation might mediate CRC metastasis. We identify the serine/threonine protein kinase IKKε as a novel and critical regulator of invadopodia formation. Furthermore, we showed that IKKε induces invadopodia formation and activity by phosphorylating kindlin-2 at S159, thus promoting metastasis in CRC.

Many kinases, which often act as signal transducers in cellular signaling cascades, have been implicated in regulating invadopodia formation 36. It has been well established that tyrosine kinases, such as Src and Arg, promote invadopodia formation by phosphorylating a number of substrates, including Tks5, WASP and cortactin 37-39; less is known about the role of serine kinase in invadopodia formation. Although the serine/threonine protein kinase IKKε has been recently found to be associated with metastasis of cancer 20, 21, 40, whether IKKε mediated invadopodia formation was previously unknown.

We found that IKKε co-localizes in invadopodia along with F-actin and the invadopodia marker Tks5, and its kinase activity is essential for regulation of invadopodia formation and ECM degradation. Similar to IKKε, other serine kinase such as protein kinase D (PKD) and p21 activated kinase 1 (Pak1) also localize in invadopodia 41, 42. PKD and Pak1 regulates invadopodia formation by phosphorylating cortactin. In this study, we identified the focal adhesion protein kindlin-2, as a novel IKKε substrate: IKKε binds to and then phosphorylates kindlin-2 at S159. It is interesting to note that although IKKε and TBK1 are very closely non-canonical IKK related kinases, kindlin-2 S159 phosphorylation was markedly reduced only when IKKε was knocked down, but not TBK1. Actually, distinct substrates and functions for TBK1 and IKKε have been disclosed 43, 44. For example, IKKε was found to phosphorylate and activate nuclear factor of activated T cells transcription factor (NFATc1) to suppress T cell activation. But knockdown of TBK-1 has no impact on NFATc1 phosphorylation and activation 43. IKKε but not TBK-1 was found to play a predominant role in innate antiviral immunity by phosphorylating of transcription factor YAP 44. Invadopodia formation in CRC cells is inhibited by kindlin-2 deletion, but rescued upon reconstitution with wild-type or phosphor-mimetic (S159D) kindlin-2. This finding is consistent with another study performed in breast cancer, in which the kindlin-2 serine phosphorylation was found to be required for invadopodia formation in breast cancer cells 30. Importantly, we observed that overexpression of IKKε did not rescue the reduced invadopodia formation in CRC cells after kindlin-2 depletion, indicating that IKKε-mediated kindlin-2 serine phosphorylation contributes to the formation of invadopodia induced by IKKε.

A number of focal adhesion proteins, such as β1-integrin and talin, have been identified to localize in invadopodia and contribute to invadopodia formation in cancer cells 45, 46. It is thus considered that invadopodia preferentially initiate at sites of focal adhesion that link the cells and ECM. Furthermore, adhesion rings surround invadopodia shortly after formation and correlate strongly with invadopodium activity 47. It is likely, therefore, that focal adhesion proteins are recruited into adhesional rings to help anchor invadopodia formation at early stages 3, 48. The recruitment of actin regulatory proteins, such as N-WASp, Arp2/3 complex and cofilin, to primarily branched F-actin core marks early-stage invadopodia formation 11, 38. Since kindlin-2 recruits Arp2/3 to promote membrane protrusions during initial cell spreading 49, 50, it is rational to speculate that IKKε-mediated kindlin-2 S159 phosphorylation might regulate Arp2/3 recruitment to early invadopodia and promote subsequent invadopodia formation.

Kindlin-2 is also phosphorylated at Y193 by Src kinase; this event might increase the kindlin-2 binding to migfilin, which in turn promotes migfilin recruitment to the focal adhesions and regulates cell migration 51, 52. Kindlin-2 and migfilin co-localize in invadopodia, and this co-localization is proposed to be affected by kindlin-2 serine phosphorylation levels 30. Thus, another possible downstream pathway regulated by IKKε-mediated kindlin-2-S159 phosphorylation is the recruitment of migfilin to facilitate invadopodia formation.

Our data clearly show that IKKε knockdown leads to a significant decrease in cell migration, invadopodia formation and invasion. This reminiscent of the nonreceptor tyrosine kinase Pyk2, which has been recently found to regulate both migration and invasion of breast cancer cells 53. Pyk2 has been shown to localize both in focal adhesion and invadopodia, which might underlie its dual role in migration and invasion 53. Although it remains to be determined whether IKKε is also localize in focal adhesions, its substrate kindlin-2 does localize in both focal adhesions and invadopodia 30, 52. It has been reported that lipoma preferred partner (LPP) also localizes in focal adhesion and invadopodia, and LPP phosphorylation by Src might switch LPP function from having a pro-migratory role in focal adhesions to a pro-invasive role in invadopodia 9. Because kindlin-2 is a key protein that couples cell focal adhesions with the induction of membrane protrusions 50, it is rational to speculate that IKKε-mediated kindlin-2 phosphorylation might also regulate a similar switch in kindlin-2 function as seen in LPP.

A growing body of evidence has shown that interventions on the proteins regulating invadopodia formation and function can markedly diminish the metastatic potential of a number of cancer cell types 45, 54, 55. Here, we found that IKKε knockdown dramatically reduces invadopodia formation and metastasis of CRC cells both *in vitro* and *in vivo*. Interestingly, the influence of IKKε knockdown on tumor metastasis *in vivo* is much more striking than its impact on cell migration *in vitro*. Actually, in the orthotopic implantation experiment, we found that the original tumors in cecum of mice carrying shIKKε cells group is less than that in mice carrying shNC cells (Supplemental figure S4). This finding indicated that tumors growth speed in mice inoculated with IKKε-knockdown CRC cells was inhibited to some degree. Therefore, we cannot rule out the possibility that the effect of IKKε knockdown on cell growth and proliferation might partly contribute to the suppression in development of tumors in metastatic sites *in vivo*. In addition, adhesion of circulating tumor cells is an important factor in formation of distant metastasis 56, 57. IKKε phosphorylates kindlin-2, which is a key molecular that regulates cell adhesion to extracellular matrix or surrounding cells. Thus, another possibility is that IKKε knockdown might influence the adhesion of circulating tumor cells to blood vessel during the process of extravasation *in vivo*. However, in this study, we provided strong evidence that IKKε knockdown effectively inhibits invadopodia formation and ECM degradation in CRC cells both *in vitro* and *in vivo*. We also demonstrated that IKKε knockdown suppresses the migratory and invasive capacity of CRC cells both *in vitro* and *in vitro*. These observations support the role of IKKε in linking invadopodia formation to CRC metastasis, and further strengthen the impact of invadopodia formation on metastasis progression. Previously, IKKε was found to promote metastasis by controlling the expression of genes involved in cellular invasion and metastasis, and subsequently associate with poor clinical outcomes in ovarian cancer 20. IKKε is also been reported to involve in the *in vivo* metastasis of breast cancer and prostate cancer through inhibiting the NF-kappaB signaling 21, 40. To the best of our knowledge, ours is the first study to reveal the real impact of IKKε on CRC metastasis. Most importantly, we provide evidence for a positive association between IKKε and kindlin-2 phosphorylation in human CRC samples, which clearly support for a direct and functional associations between IKKε and kindlin-2 phosphorylation in human CRC tissues. Our findings reveal the crucial role of the IKKε-kindlin-2 axis in linking invadopodia formation to CRC metastasis. Considering the role of kindlin-2 in regulating focal adhesion and invadopodia, whether IKKε also localizes to focal adhesions and modulates cytoskeleton remodeling in membrane structures await further investigation.

In conclusion, our data demonstrate the novel role of IKKε in regulating invadopodia formation and provide mechanistic insight into the regulation of adhesive structures involved in cell metastasis. We believe that these findings implicate the therapeutic potential of IKKε to suppress CRC metastasis.

## Supplementary Material

Supplementary figures and tables.

## Abbreviations

CRC: Colorectal cancer
H&E: Hematoxylin and eosin
IHC: Immunohistochemical analysis
IP: Immunoprecipitation
DMSO: dimethyl sulfoxide
IPTG: Isopropy-β-D-thiogalactoside
ECM: extracellular matrix
CK18: cytokeratin 18
EpCAM: epithelial cell adhesion molecule

## References

[1] Bray F, Ferlay J, Soerjomataram I, Siegel RL, Torre LA, Jemal A (2018). Global cancer statistics 2018: GLOBOCAN estimates of incidence and mortality worldwide for 36 cancers in 185 countries. CA Cancer J Clin.

[2] Chiang AC, Massague J (2008). Molecular basis of metastasis. N Engl J Med.

[3] Eddy RJ, Weidmann MD, Sharma VP, Condeelis JS (2017). Tumor Cell Invadopodia: Invasive Protrusions that Orchestrate Metastasis. Trends Cell Biol.

[4] Murphy DA, Courtneidge SA (2011). The 'ins' and 'outs' of podosomes and invadopodia: characteristics, formation and function. Nat Rev Mol Cell Biol.

[5] Yamaguchi H (2012). Pathological roles of invadopodia in cancer invasion and metastasis. Eur J Cell Biol.

[6] Ren XL, Qiao YD, Li JY, Li XM, Zhang D, Zhang XJ (2018). Cortactin recruits FMNL2 to promote actin polymerization and endosome motility in invadopodia formation. Cancer Lett.

[7] Leong HS, Robertson AE, Stoletov K, Leith SJ, Chin CA, Chien AE (2014). Invadopodia are required for cancer cell extravasation and are a therapeutic target for metastasis. Cell Rep.

[8] Eckert MA, Lwin TM, Chang AT, Kim J, Danis E, Ohno-Machado L (2011). Twist1-induced invadopodia formation promotes tumor metastasis. Cancer Cell.

[9] Ngan E, Stoletov K, Smith HW, Common J, Muller WJ, Lewis JD (2017). LPP is a Src substrate required for invadopodia formation and efficient breast cancer lung metastasis. Nat Commun.

[10] Jeannot P, Nowosad A, Perchey RT, Callot C, Bennana E, Katsube T (2017). p27Kip1 promotes invadopodia turnover and invasion through the regulation of the PAK1/Cortactin pathway.

[11] Yamaguchi H, Lorenz M, Kempiak S, Sarmiento C, Coniglio S, Symons M (2005). Molecular mechanisms of invadopodium formation: the role of the N-WASP-Arp2/3 complex pathway and cofilin. J Cell Biol.

[12] Boehm JS, Zhao JJ, Yao J, Kim SY, Firestein R, Dunn IF (2007). Integrative genomic approaches identify IKBKE as a breast cancer oncogene. Cell.

[13] Guan H, Zhang H, Cai J, Wu J, Yuan J, Li J (2011). IKBKE is over-expressed in glioma and contributes to resistance of glioma cells to apoptosis via activating NF-kappaB. J Pathol.

[14] Cheng A, Guo J, Henderson-Jackson E, Kim D, Malafa M, Coppola D (2011). IkappaB Kinase epsilon expression in pancreatic ductal adenocarcinoma. Am J Clin Pathol.

[15] Guo JP, Shu SK, He L, Lee YC, Kruk PA, Grenman S (2009). Deregulation of IKBKE is associated with tumor progression, poor prognosis, and cisplatin resistance in ovarian cancer. Am J Pathol.

[16] Wang Y, Lu X, Zhu L, Shen Y, Chengedza S, Feng H (2013). IKK epsilon kinase is crucial for viral G protein-coupled receptor tumorigenesis. Proc Natl Acad Sci U S A.

[17] Barbie TU, Alexe G, Aref AR, Li S, Zhu Z, Zhang X (2014). Targeting an IKBKE cytokine network impairs triple-negative breast cancer growth. J Clin Invest.

[18] Hutti JE, Shen RR, Abbott DW, Zhou AY, Sprott KM, Asara JM (2009). Phosphorylation of the tumor suppressor CYLD by the breast cancer oncogene IKKepsilon promotes cell transformation. Mol Cell.

[19] Guo JP, Tian W, Shu S, Xin Y, Shou C, Cheng JQ (2013). IKBKE phosphorylation and inhibition of FOXO3a: a mechanism of IKBKE oncogenic function. PLoS One.

[20] Hsu S, Kim M, Hernandez L, Grajales V, Noonan A, Anver M (2012). IKK-epsilon coordinates invasion and metastasis of ovarian cancer. Cancer Res.

[21] Cheng C, Ji Z, Sheng Y, Wang J, Sun Y, Zhao H (2018). Aphthous ulcer drug inhibits prostate tumor metastasis by targeting IKKvarepsilon/TBK1/NF-kappaB signaling. Theranostics.

[22] Seals DF, Azucena EF Jr, Pass I, Tesfay L, Gordon R, Woodrow M (2005). The adaptor protein Tks5/Fish is required for podosome formation and function, and for the protease-driven invasion of cancer cells. Cancer Cell.

[23] Ayala I, Baldassarre M, Giacchetti G, Caldieri G, Tete S, Luini A (2008). Multiple regulatory inputs converge on cortactin to control invadopodia biogenesis and extracellular matrix degradation. J Cell Sci.

[24] Mader CC, Oser M, Magalhaes MA, Bravo-Cordero JJ, Condeelis J, Koleske AJ (2011). An EGFR-Src-Arg-cortactin pathway mediates functional maturation of invadopodia and breast cancer cell invasion. Cancer Res.

[25] Lin J, Lin W, Ye Y, Wang L, Chen X, Zang S (2017). Kindlin-2 promotes hepatocellular carcinoma invasion and metastasis by increasing Wnt/beta-catenin signaling. J Exp Clin Cancer Res.

[26] Yang JR, Pan TJ, Yang H, Wang T, Liu W, Liu B (2016). Kindlin-2 promotes invasiveness of prostate cancer cells via NF-kappaB-dependent upregulation of matrix metalloproteinases. Gene.

[27] Shen Z, Ye Y, Dong L, Vainionpaa S, Mustonen H, Puolakkainen P (2012). Kindlin-2: a novel adhesion protein related to tumor invasion, lymph node metastasis, and patient outcome in gastric cancer. Am J Surg.

[28] Wei X, Wang X, Zhan J, Chen Y, Fang W, Zhang L (2017). Smurf1 inhibits integrin activation by controlling Kindlin-2 ubiquitination and degradation. J Cell Biol.

[29] Li H, Deng Y, Sun K, Yang H, Liu J, Wang M (2017). Structural basis of kindlin-mediated integrin recognition and activation. Proc Natl Acad Sci U S A.

[30] Artym VV, Swatkoski S, Matsumoto K, Campbell CB, Petrie RJ, Dimitriadis EK (2015). Dense fibrillar collagen is a potent inducer of invadopodia via a specific signaling network. J Cell Biol.

[31] Ran FA, Hsu PD, Wright J, Agarwala V, Scott DA, Zhang F (2013). Genome engineering using the CRISPR-Cas9 system. Nat Protoc.

[32] Tang M, Li Z, Zhang C, Lu X, Tu B, Cao Z (2019). SIRT7-mediated ATM deacetylation is essential for its deactivation and DNA damage repair. Sci Adv.

[33] Li Z, Li Y, Tang M, Peng B, Lu X, Yang Q (2018). Destabilization of linker histone H1.2 is essential for ATM activation and DNA damage repair. Cell Res.

[34] Liu C, Yang Q, Zhu Q, Lu X, Li M, Hou T (2020). CBP mediated DOT1L acetylation confers DOT1L stability and promotes cancer metastasis. Theranostics.

[35] Reilly SM, Chiang SH, Decker SJ, Chang L, Uhm M, Larsen MJ (2013). An inhibitor of the protein kinases TBK1 and IKK-varepsilon improves obesity-related metabolic dysfunctions in mice. Nat Med.

[36] Foxall E, Pipili A, Jones GE, Wells CM (2016). Significance of kinase activity in the dynamic invadosome. Eur J Cell Biol.

[37] Diaz B, Shani G, Pass I, Anderson D, Quintavalle M, Courtneidge SA (2009). Tks5-dependent, nox-mediated generation of reactive oxygen species is necessary for invadopodia formation. Sci Signal.

[38] Oser M, Yamaguchi H, Mader CC, Bravo-Cordero JJ, Arias M, Chen X (2009). Cortactin regulates cofilin and N-WASp activities to control the stages of invadopodium assembly and maturation. J Cell Biol.

[39] Lapetina S, Mader CC, Machida K, Mayer BJ, Koleske AJ (2009). Arg interacts with cortactin to promote adhesion-dependent cell edge protrusion. J Cell Biol.

[40] Bishop RT, Marino S, de Ridder D, Allen RJ, Lefley DV, Sims AH (2019). Pharmacological inhibition of the IKKepsilon/TBK-1 axis potentiates the anti-tumour and anti-metastatic effects of Docetaxel in mouse models of breast cancer. Cancer Lett.

[41] Moshfegh Y, Bravo-Cordero JJ, Miskolci V, Condeelis J, Hodgson L (2014). A Trio-Rac1-Pak1 signalling axis drives invadopodia disassembly. Nat Cell Biol.

[42] De Kimpe L, Janssens K, Derua R, Armacki M, Goicoechea S, Otey C (2009). Characterization of cortactin as an in vivo protein kinase D substrate: interdependence of sites and potentiation by Src. Cell Signal.

[43] Zhang J, Feng H, Zhao J, Feldman ER, Chen SY, Yuan W (2016). IkappaB Kinase epsilon Is an NFATc1 Kinase that Inhibits T Cell Immune Response. Cell Rep.

[44] Wang S, Xie F, Chu F, Zhang Z, Yang B, Dai T (2017). YAP antagonizes innate antiviral immunity and is targeted for lysosomal degradation through IKKvarepsilon-mediated phosphorylation. Nat Immunol.

[45] Beaty BT, Wang Y, Bravo-Cordero JJ, Sharma VP, Miskolci V, Hodgson L (2014). Talin regulates moesin-NHE-1 recruitment to invadopodia and promotes mammary tumor metastasis. J Cell Biol.

[46] Beaty BT, Sharma VP, Bravo-Cordero JJ, Simpson MA, Eddy RJ, Koleske AJ (2013). beta1 integrin regulates Arg to promote invadopodial maturation and matrix degradation. Mol Biol Cell.

[47] Branch KM, Hoshino D, Weaver AM (2012). Adhesion rings surround invadopodia and promote maturation. Biol Open.

[48] Maziveyi M, Dong S, Baranwal S, Alahari SK (2018). Nischarin regulates focal adhesion and Invadopodia formation in breast cancer cells. Mol Cancer.

[49] Theodosiou M, Widmaier M, Bottcher RT, Rognoni E, Veelders M, Bharadwaj M (2016). Kindlin-2 cooperates with talin to activate integrins and induces cell spreading by directly binding paxillin. Elife.

[50] Bottcher RT, Veelders M, Rombaut P, Faix J, Theodosiou M, Stradal TE (2017). Kindlin-2 recruits paxillin and Arp2/3 to promote membrane protrusions during initial cell spreading. J Cell Biol.

[51] Liu Z, Lu D, Wang X, Wan J, Liu C, Zhang H (2015). Kindlin-2 phosphorylation by Src at Y193 enhances Src activity and is involved in Migfilin recruitment to the focal adhesions. FEBS Lett.

[52] Tu Y, Wu S, Shi X, Chen K, Wu C (2003). Migfilin and Mig-2 link focal adhesions to filamin and the actin cytoskeleton and function in cell shape modulation. Cell.

[53] Genna A, Lapetina S, Lukic N, Twafra S, Meirson T, Sharma VP (2018). Pyk2 and FAK differentially regulate invadopodia formation and function in breast cancer cells. J Cell Biol.

[54] Wu Q, Chen D, Luo Q, Yang Q, Zhao C, Zhang D (2018). Extracellular matrix protein 1 recruits moesin to facilitate invadopodia formation and breast cancer metastasis. Cancer Lett.

[55] Kuo TL, Cheng KH, Shan YS, Chen LT, Hung WC (2019). beta-catenin-activated autocrine PDGF/Src signaling is a therapeutic target in pancreatic cancer. Theranostics.

[56] Haier J, Nicolson GL (2001). The role of tumor cell adhesion as an important factor in formation of distant colorectal metastasis. Dis Colon Rectum.

[57] Xie J, Zhao R, Gu S, Dong H, Wang J, Lu Y (2014). The architecture and biological function of dual antibody-coated dendrimers: enhanced control of circulating tumor cells and their hetero-adhesion to endothelial cells for metastasis prevention. Theranostics.

