# Supplementary material for: IKKε phosphorylates kindlin-2 to induce invadopodia formation and promote colorectal cancer metastasis
Source: Theranostics. 2020 Jan 16;10(5):2358–73. doi: 10.7150/thno.40397 (PMC7019159; doi:10.7150/thno.40397)
Supplement: Supplementary file 1 — Supplementary figures and tables. [file thnov10p2358s1.pdf]

Supplementary figures

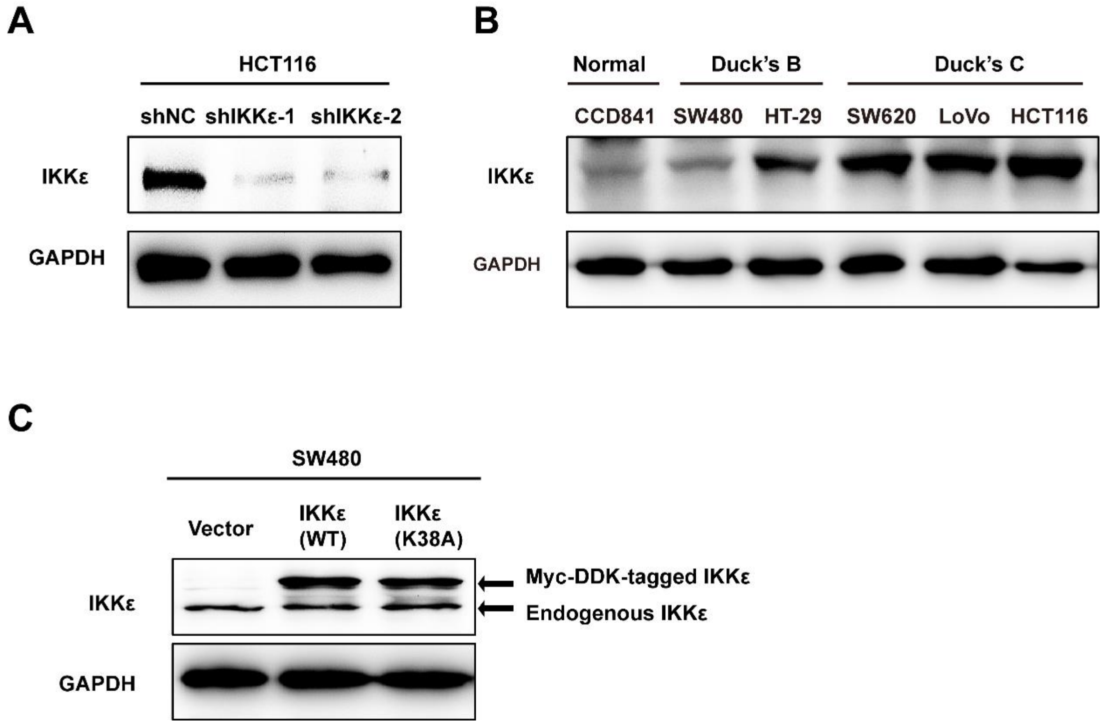

Figure S1. Expression levels of IKKε in variety of colon cancer cell lines.

(A) Western blot analysis of lysates from HCT116 cells stably transfected with non-targeting shRNA or shRNA targeting IKKε. (B) IKKε protein levels in the indicated cells were assessed by western blotting. (C) Western blot analysis of lysates from SW480 cells stably transfected with Myc-DDK-tagged wild-type IKKε (WT) or mutant IKKε (K38A).

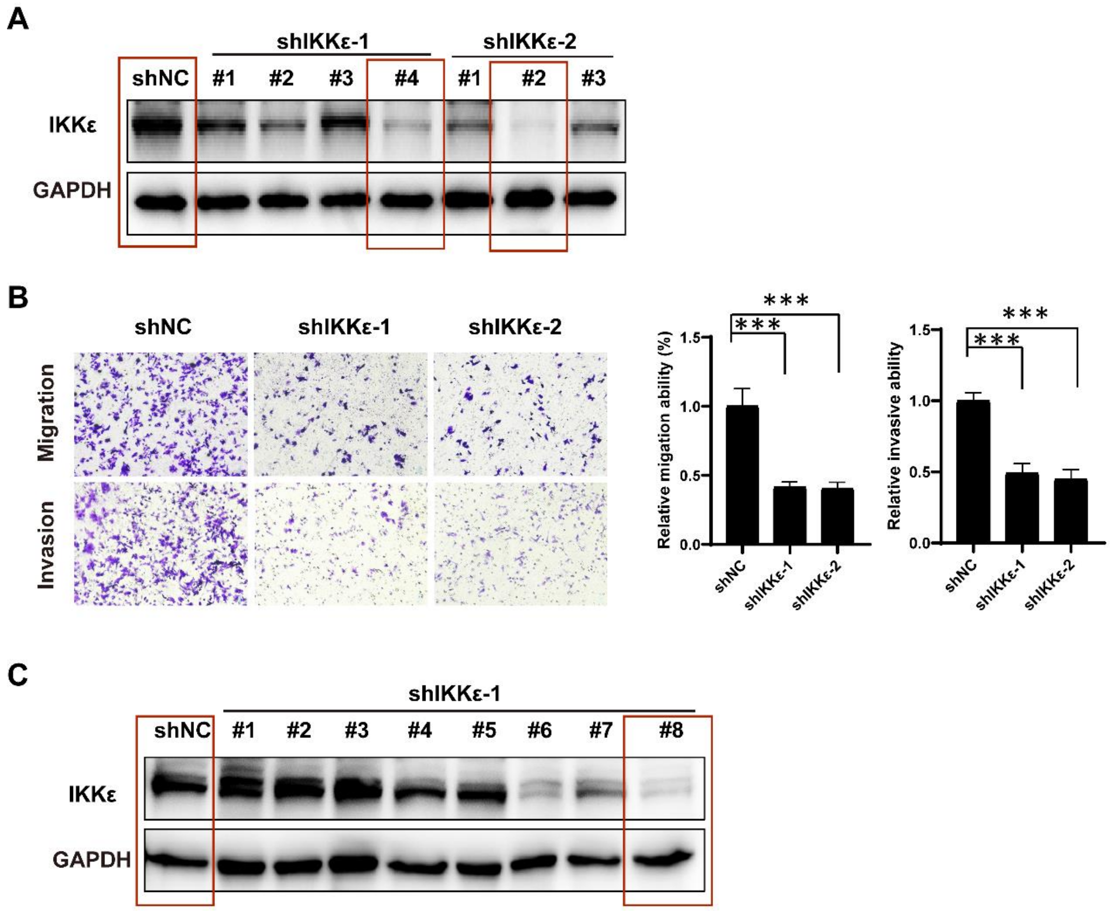

Figure S2. Confirmation of expression levels of IKKε and the migrative/invasive ability in IKKε-knockdown in CRC cells.

(A) Lovo cells stably expressed luciferase and IKKε-specific shRNA or control shRNA. Total protein was isolated from the indicated cells and examined for IKKε protein levels by western blotting. The red boxes highlight the cells used in the experiments. (B) Transwell invasion migration assay (top) and invasion assay (bottom) for Lovo cells stably transfected with non-targeting shRNA or shRNA targeting IKKε (left). The relative migration and invasive ability was normalized to shNC (right). All data represent the means ± S.D. of three independent experiments (****P* < 0.001). (C) HCT116 cells stably expressed luciferase and IKKε-specific shRNA or control shRNA. Total protein was isolated from the indicated cells and examined for IKKε protein levels by western blotting. The red boxes highlight the cells used in the experiments.

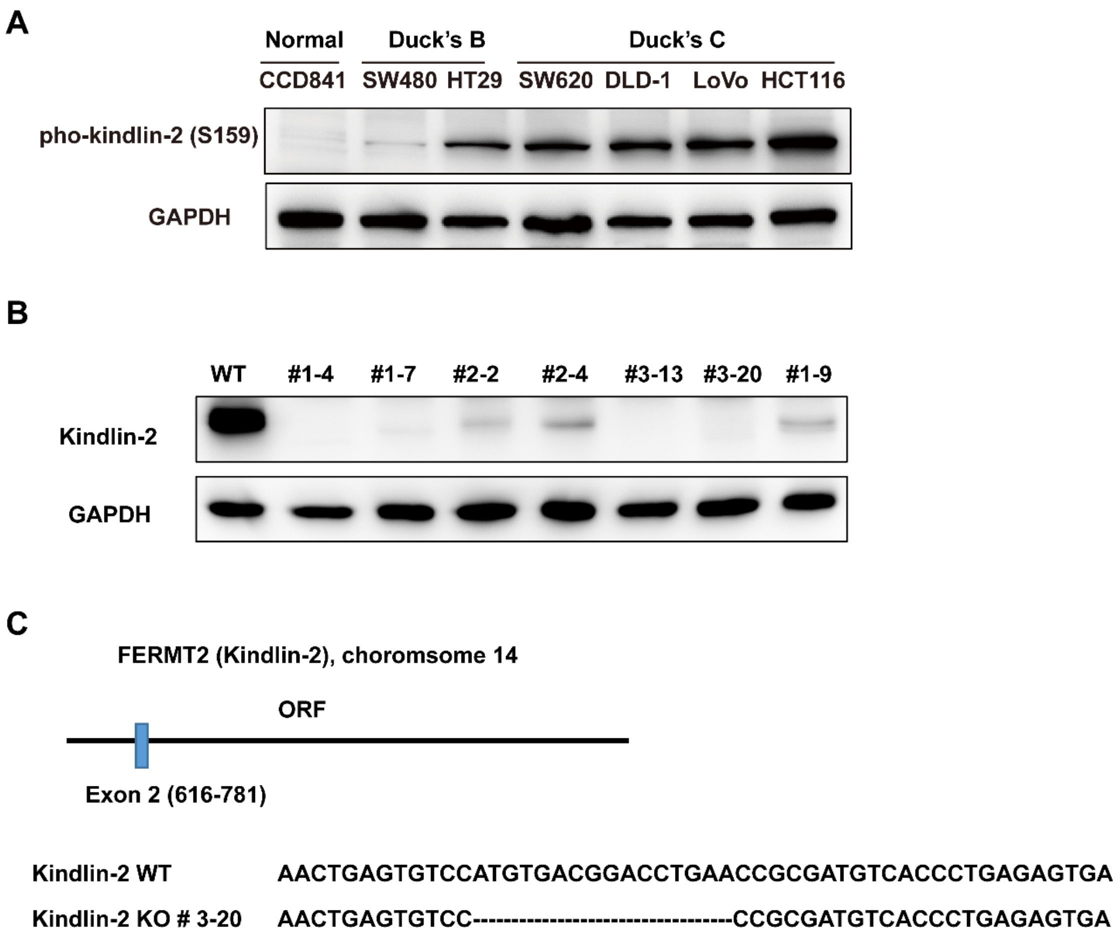

Figure S3. Confirmation of kindlin-2-knockout in HCT116 cells.

(A) Phosphorylated-kindlin-2(S159) protein levels in the indicated cells were assessed by western blotting. (B) Kindlin-2 expression levels in HCT116 clones generated from cells that were stably transfected with the sgRNA-Cas9 knockout vector. (C) Sequence of the region of kindlin-2 that misses bases in clone #3-20 of the stable CRISPR/Cas9 knockout HCT116 cells.

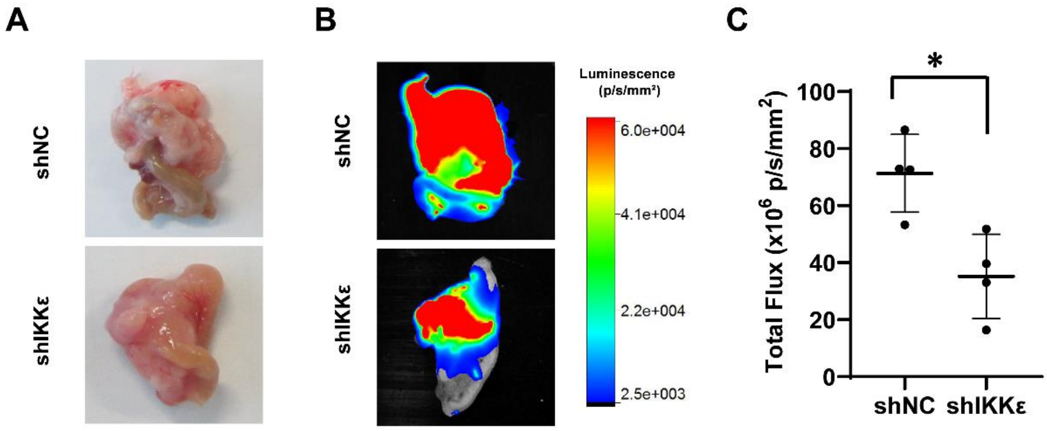

Figure S4. The growth of tumors in cecum of mice orthotopically microinjected with IKKε-knockdown CRC cells was inhibited.

(A) The gross view of tumor nodules established in cecum and (B) the bioluminescence images of cecum from mice orthotopically microinjected with HCT116 cells stably expressing shNC or shIKKε. (C) Quantitative analysis of photon flux in shNC (n=4) and shIKKε (n=4) mice (*P<0.05).

Supplementary table

Table. S1 Correlation between IKKε expression and clinicopathological characteristics

	variables	IKKε expression		total	χ2	p value
		low	High			
Age (year)					0.02	0.889
	≤68	69	28	97		
	＞68	66	28	94		
	null					
T stage					0.232	0.63
	T1/T2	7	2	9		
	T3/T4	123	52	175		
TNM stage					12.8	0.000
	I/II	78	17	95		
	III/IV	51	37	88		
	null					
N stage					10.3	0.001
	N0	82	20	102		
	N1/N2	50	35	85		
	null					
M stage					1.067	0.302
	M0	131	52	183		
	M1	5	4	9		
	null					
Sex					0.112	0.738
	Female	59	23	82		
	Male	76	33	109		
	Null					
grade					3.35	0.067
	I/II	115	41	156		
	III	21	15	36		

Pearson’s chi-square tests were used to analyze the correlation between IKKε and clinical features. Results were considered statistically significant at $P<0.05$.
